# Inventory analysis and environmental life cycle impact assessment of hotel food waste management for bio-circular economy development in Zimbabwe

**DOI:** 10.1007/s10661-024-13314-6

**Published:** 2024-11-14

**Authors:** Trust Nhubu, Charles Mbohwa

**Affiliations:** 1 College of Graduate Studies, School of Interdisciplinary Research and Graduate Studies (SIRGS), University of South Africa Pretoria Campus, Pretoria, South Africa; 2 College of Science, Engineering and Technology, School of Engineering, Department of Industrial Engineering, University of South Africa Florida Campus, Johannesburg, South Africa

**Keywords:** Food waste, Environmental life cycle assessment, Landfill, Composting, Anaerobic digestion, Hospitality industry

## Abstract

This study is an inventory analysis and environmental life cycle assessment of hotel food waste management that seeks to inform efforts towards the development of bio circular economy in Zimbabwe. An audit of food waste generation and an inventory analysis of the prevailing food waste management practices at three selected hotels was undertaken. The greenhouse gas emissions from the prevailing disposal of food waste at dumpsites and the proposed biological treatment of food waste were evaluated using the Tier 1 FOD method and Tier 1 method of biological treatment using the 2019 refined 2006 Intergovernmental Panel on Climate Change guidelines. Environmental life cycle assessment was also conducted for the open dumping, composting, and anaerobic digestion. The average food waste generation within the Zimbabwean hospitality industry was estimated at 1.63 kg/guest/day, with a minimum and maximum of 1.01 and 2.25 kg/guest/day, respectively. Source-separated food waste is currently being collected indiscriminately by municipal waste collection trucks for final disposal at the official landfills or dumpsites. This calls for the need for an offtake system of the source-separated food waste in the form of composting or anaerobic digestion. Study results showed that the disposal of food waste at solid waste disposal sites contributes to the highest greenhouse gas emissions, followed by composting with a 75% reduction in greenhouse emissions. Anaerobic digestion brings about a maximum reduction in greenhouse emissions of 97%. Environmental life cycle assessment results also show that anaerobic digestion is the best method leading to net negative environmental impacts.

**Supplementary Information:**

The online version contains supplementary material available at 10.1007/s10661-024-13314-6.

## Introduction

Tourism has become one of the fastest-growing sectors globally, resulting in increased municipal solid waste (MSW) generation, especially food waste (FW). FW constitutes more than 60% of the MSW generated within the hospitality industry. FW generated from the hospitality industry constitutes 9% of the FW generated by the food sector globally (Pirani & Arafat, 2016). The generation of FW has multiple socioeconomic and environmental implications. UNEP (2024) reported that FW generation is regarded as both a market and an environmental failure. This is so because it results in the dumping of FW worth more than US$1 trillion annually and contributes between 8 and 10% of greenhouse gas (GHG) emissions. Pirani and Arafat (2014) noted that the environmental impacts of the hospitality industry thus partly manifest from the corresponding FW generation and management.

The considerable resource-intensive nature of food production is attributable to the environmental impacts of FW. Silvennoinen et al. (2014) reported that an estimated 250 km^3^, 70% of the Earth’s infinite and vulnerable freshwater resources, is used in food production per annum. Food production leads to habitat loss as tracts of land are converted to agricultural land, with an estimated 1.4 × 10^9^ ha constituting 28% of the total land area globally being used for food production. Apart from land and water, food production, distribution, and storage use materials and energy, which bring about increased environmental loads along the food value chain and life cycle. FW thus translates to the loss of all these resources that would have been used in its production and along the value chain.

Despite more than 750 million people suffering from hunger every year, 150 million children younger than 5 years have essential nutrients constrained diets, leading to stunted growth and development (UNEP, 2024). A reduction in the prevailing FW generation rates will contribute to the attainment of Sustainable Development Goal (SDG) 12. Hence, target 12.3 is important under SDG 12, which provides for a 50% reduction in global FW per capita by retailers and consumers together with a reduction in food losses across food supply chains by 2030. The contribution of FW and its management to the attainment of SDGs is provided in Table 1.

The socioeconomic and environmental impacts associated with FW generation and management have rendered FW generation a global challenge because of the increased attention given to the need to implement appropriate FW reduction and prevention, valorization pathways, and management systems that could address these impacts (Lin et al., 2022; Thi et al., 2015; Woon et al., 2021). Life cycle assessment (LCA) methodologies evaluate the economic and environmental impacts of various FW management practices, including transportation, reduction, prevention, treatment, and disposal including the potential recovery of materials and energy. Environmental life cycle assessment (ELCA) has matured as a tool for assessing FW management-associated environmental and human health impacts. Several LCA studies have been undertaken with Batool et al. (2024) having critically reviewed ELCA studies that assessed the environmental impacts of landfilling, anaerobic digestion (AD), composting, hydrothermal carbonization, and gasification of FW.

Zimbabwe has a national integrated solid waste management plan which came into effect in 2014 whose implementation has remained stagnant. The plan lacked baseline information regarding the potential reductions in GHG emissions as well as other environmental and human health impacts that come with the proposed composting or AD of biodegradable waste including FW. The Zimbabwe Long Term Low Emissions Development Strategy (LT-LEDS) and nationally determined contribution (NDC) (Zimbabwe Revised Nationally Determined Contribution, 2021) also provide for potential reductions in GHG emissions from the management of biodegradable MSW fractions through composting and AD. However, the LT-LEDS and NDC-provided GHG reductions are based on the entire waste sector-wide proposals at the local authority level (municipal or town). The proposals do not consider the various sub-sectors that generate biodegradable waste such as hotels. Such institutions like hotels have systems and structures that allow for ease implementation of source separation of biodegradables which aids the design and operation of composting and AD systems.

This study seeks to inform the design and development of low-emission and sustainable FW management systems specifically focusing on the hospitality industry in Zimbabwe. This study assessed the environmental impacts of the current FW management practice (dumping at open dumpsites) at three selected hotels and compared them with the impacts of composting and anaerobic digestion using ELCA. In addition, the impacts of ELCA-derived global warming on open dumping, composting, and anaerobic digestion were compared with those estimated from the Intergovernmental Panel on Climate Change (IPCC) guidelines. Study findings will aid the implementation of Zimbabwe’s Long Term Low Emissions Development Strategy (LT-LEDS), nationally determined contribution (NDC) as well as raise awareness on the importance of sustainable FW management. The Zimbabwe national integrated solid waste management plan is due for review; hence, study findings will contribute to the review process.

**Table 1:** Contribution of FW and its management to the attainment of SDGs

SDG	Applicable target	Description
2	2.1	Avoidance and reduction of FW can contribute to the target to end hunger and ensure food accessibility by all people including the poor and those facing vulnerabilities by 2030
	2.2	The generation of FW militates against the target to end all forms of malnutrition by 2030 as well as the fight to end stunted growth in children below 5 years of age and nutritional challenges for adolescent girls, pregnant and lactating women, and older persons by 2025
6	6.2	The proper management of FW through AD and composting promotes improved sanitation and hygiene considering the environmental challenges from a sanitation and hygiene perspective that associated with the improper FW management and disposal
7	7.1	Generation of renewable energy (biogas) from the AD of FW that could be used for heating and cooking, combine heat and power generation, upgraded to vehicular fuel is part of global efforts towards ensuring the universal access to affordable, reliable, and modern energy services by 2030
	7.2	The use of biogas from the AD of FW will significantly increase the renewable energy share in national, regional, and global energy mix by 2030
12	12.3	The adoption of sustainable consumption strategies at hospitality institutions will contribute to the global efforts to have 50% per capita global FW reduction at the retail and consumer levels by 2030
	12.4	Though the target year was 2020, AD and composting of FW will contribute to the attainment of environmentally sound management of FW resultantly reducing the associated air, water, and soil pollution and their corresponding human health and environmental impacts

## Materials and methods

### Inventory analysis of FW management practices

An audit of waste generation and an inventory analysis of the prevailing waste management practices at the three selected hotels were performed between November and December 2023. The period is regarded as a high peak period characterized by high tourism activity and number of tourists. Audit results provide the maximum probable scenario regarding FW generation within the selected hotels. Although waste generation varies across different temporal scales (weekday, week of month, and month of year) and spatial scales or localities which highlight the need for longitudinal yearlong sampling and waste generation data measurements or audits (Abel, 2007), time, financial and human resources constrained the undertaking of audits outside the period between November 2023 and December 2023. Daily measurements of the various waste fractions mainly food, plastic, paper, and tins were recorded using a scale for over 5 days. The total and average waste generated were computed and correlated to the hotel room occupancy. A 2 kg sugar sample was used to calibrate and ensure the accuracy of the weighing scale. It was assumed that the respective hotel operations are standardized with insignificant variations in their food value chain and in the behavior of hotel guests regarding eating habits and FW generation. The annual average FW generation for the respective hotel (PAx) was computed from Eq. 1:

1 $$P_{Ax}=\frac{{RO}_{Ax}}{{R0}_{ax}}\left(P_{ax}\right)$$

where RO_Ax_ is the annual average percentage room occupancy for hotel x, RO_ax_ is the observed room occupancy during the audit, and P_ax_ is the estimated daily FW generation during the audit at hotel x in kg.

### GHG emission estimation using IPPC guidelines

Methane (CH_4_) emissions from the disposal of FW generated at the hotels in solid waste disposal sites (SWDS) were estimated using the Tier 1 First Order Decay (FOD) method of the 2019 refined 2006 IPCC guidelines. This method was also used to estimate the waste sector emissions reported in Zimbabwe’s Fourth National Communication to the United Nations Framework Convention on Climate Change (UNFCCC), which includes a 50-year timespan for all the food waste to decompose. Default IPPC parameter values were used together with actual field measurements. The respective RO_Ax_, RO_ax_, P_ax_, and P_Ax_ were used. It was assumed that a direct and positive relationship exists between solid waste generation and room occupancy levels.

GHG emissions from the composting and AD of FW were also estimated using the Tier 1 method of biological treatment following the 2019 refined 2006 IPCC guidelines. The IPCC default CH_4_ and N_2_O emission factors of 4 g CH_4_/kg and 0.24 g N_2_O/kg of waste treated were used for composting on a wet weight basis. An IPCC default CH_4_ emission factor of 0.8 g CH_4_/kg of waste treated was used for anaerobic digestion on a wet weight basis, with N_2_O emissions considered negligible. The CH_4_ and N_2_O emissions from the composting and AD of the food waste generated in Gg were estimated using Eqs. 2 and 3:

2 $${CH}_4=\sum_i{(M}_i\ast{EF}_i)\ast10^{-3}-R$$

3 $$N_2o=\sum_i{(M}_i\ast{EF}_i)\ast10^{-3}$$

where

*M*_i_: is the mass of organic waste treated by biological treatment type *i*, Gg,
*i*: is composting or anaerobic digestion,
*EF*: is the emission factor for treatment *i*, g CH_4_/kg waste treated, and
*R*: is the total amount of CH_4_ recovered under anaerobic digestion in the inventory year, Gg CH_4_, which was regarded as zero.

Upon estimation of the GHG emissions from the disposal of FW at SWDS and the treatment of FW through AD and composting, the percentage reduction in the emissions that comes with the movement from disposal of FW at SWDS to either composting or AD was calculated using Eq. 4:

4 $$y=\frac{A-B}A$$

where

*y*: is the percentage reduction in GHG emissions,
*A*: are the GHG emissions from the disposal of FW at SWDS, and
*B*: are the GHG emissions from the treatment of FW through either composting or anaerobic digestion.

The major challenge with the use of the 2019 refined 2006 IPCC guidelines is the use of IPCC default values for methane correction factor (MCF) for the management of FW in SWDS as well the IPCC default CH_4_ and N_2_O emission factors for composting and AD due to the absence of Zimbabwe specific values. The use of the IPCC default per capita waste generation had no effect on the FW generated at respective hotel institutions. This is so because the population whose product with the default per capita waste generation gives the estimated total amount of FW generated per year per institution was calculated and used.

### Environmental life cycle assessment

**Fig. 1:**
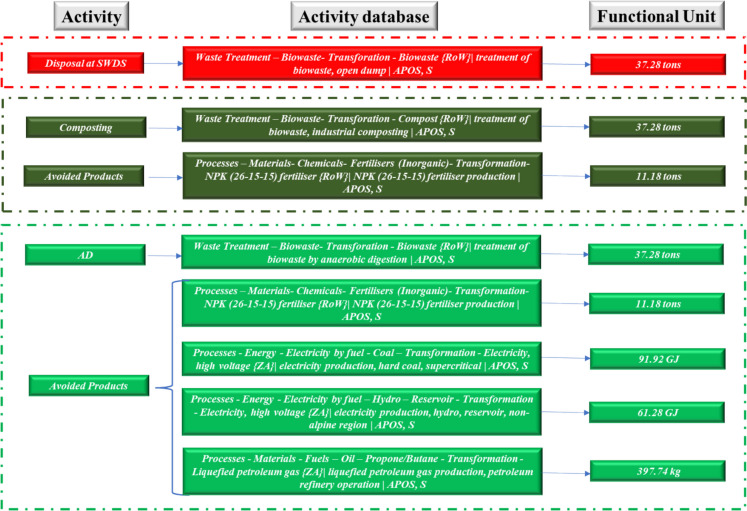
ELCA system boundary inventory and system boundary for institution 1

**Fig. 2:**
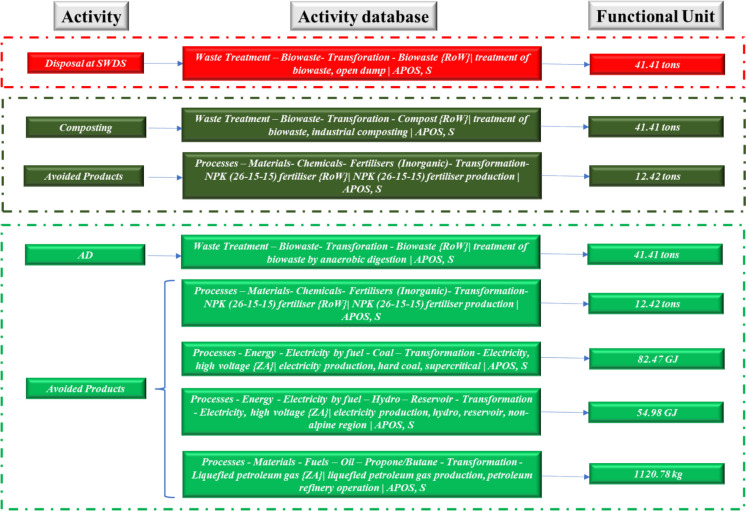
ELCA system boundary inventory and system boundary for institution 2

**Fig. 3:**
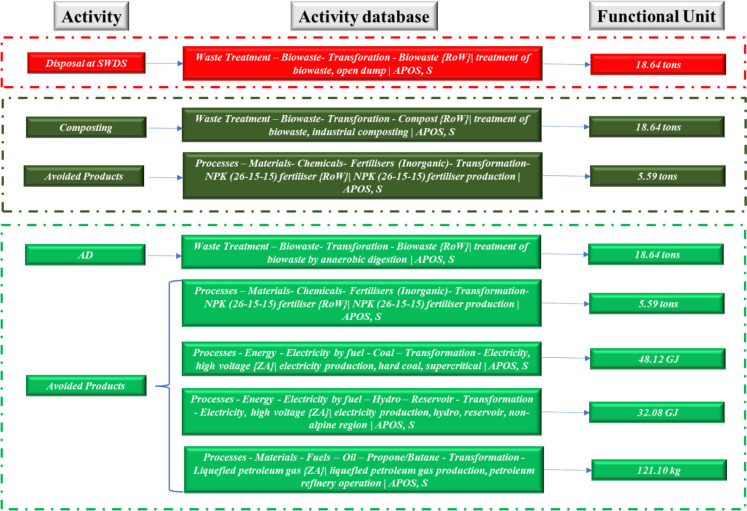
ELCA system boundary inventory and system boundary for institution 3

A life cycle assessment was conducted to assess the environmental impacts of open dumping (the prevailing management practice) and proposed composting and anaerobic digestion of FW generated at the respective hotels. SimaPro version 9.5.0.2 was used for the LCA. Impact loads of the processes and materials were collected from the Ecoinvent 3v database. The ReCiPe 2016 v1.1 method at the midpoint was used (Huijbregts et al., 2017). The annual FW generation (P_Ax_) was used as the functional unit. The SimaPro version 9.5.0.2 embedded Ecoinvent 3v databases for open dumping, AD, and composting of food waste were used in the ELCA as provided in Figs. 1, 2, and 3, respectively. The open dumping of FW starts with the final disposal of FW at a SWDS without considering the associated FW transport-related emissions. The FW-specific short- and long-term emissions to air and land through landfill gas and landfill leachate, respectively, were thus considered for simulation. AD process included the storage of biomass feedstock on arrival at the AD facility, production of biogas and digestate together with the energy generation in a combined heat and power generation unit. Composting activities included storage of biomass feedstock upon arrival at the facility, energy needs for the composting process. All infrastructure-related emissions were not included during the simulations for open dumping, composting, and AD of FW.

A functional unit of 37.28 tons of FW per annum was used at institution 1. The avoided products involved the production of 11.18 tons of chemical fertilizers for both composting and AD. A total of 171.50 GJ of energy was estimated to be generated from the 7.46 × 10^3^ m^3^ of biogas produced from the AD process at institution 1. The energy was assumed to replace grid electricity and liquefied petroleum gas (LPG) based on an energy mix of 89% grid electricity and 11% LPG that was estimated at institution 1 during the audit. Therefore, 153.20 and 18.30 GJ of grid electricity and LPG are avoided. Regarding the avoided grid electricity, 91.92 and 61.28 GJ were considered to come from coal thermal power plants and large hydro, respectively. This was based on the Zimbabwe grid electricity mix of 60% and 40%.

A functional unit of 41.41 ton of FW per annum was used at institution 2. The avoided products involved the production of 12.42 ton of chemical fertilizers for both composting and AD. A total of 190.47 GJ of energy was estimated to be avoided from the 8.28 × 10^3^ m^3^ of biogas produced from the AD process at institution 2. The energy was assumed to replace grid electricity and liquefied petroleum gas (LPG) based on an energy mix of 72% grid electricity and 28% LPG that was estimated at institution 1 during the audit. Therefore, 137.45 and 53.01 GJ of grid electricity and LPG are avoided. It was estimated that of the 137.45 GJ of the avoided grid electricity, 82.47 and 54.98 GJ are produced from coal thermal power plants and large hydro, respectively, based on the Zimbabwe grid electricity mix.

A functional unit of 18.64 ton of FW per annum was used at institution 3. The avoided products involved the production of 5.59 ton of chemical fertilizers for both composting and AD. A total of 85.76 GJ of energy was estimated to be avoided from the 3.73 × 10^3^ m^3^ of biogas produced from the AD process at institution 3. The energy was assumed to replace grid electricity and liquefied petroleum gas (LPG) based on an energy mix of 94% grid electricity and 6% LPG that was estimated at institution 1 during the audit. Therefore, 80.19 and 5.57 GJ of grid electricity and LPG are avoided. Of the 80.19 GJ avoided grid electricity, 48.12 and 32.08 GJ were assumed to be generated from coal thermal power plants and large hydro, respectively, based on the Zimbabwe grid electricity mix.

#### Sensitivity analysis

The transport inventory for the disposal of FW generated at institutions 1 and 2 at SWDS was used for sensitivity analysis. The inventory was based on the product of distance traveled by municipal waste collection trucks from the institution to the SWDS and the average FW generation. This is mathematically expressed in Eq. 5:

5 $$W=365\ast\left(\frac{y\ast P_{Ax}}{1000}\right)$$

where *y* is the distance between the institution and SWDS and *W* is the measure of FW transported in ton kilometers (tkm). The tkm of the FW transported that are provided in Table 2 were therefore used for assessing the impacts of FW transportation to the SWDS. No impacts were considered for the transportation of FW to SWDS at institution 3 since the SWDS is situated at the institution. Likewise, no FW transportation to AD and composting facilities-related impacts were assessed. This is so because the AD and composting facilities were proposed to be established within the vicinity of the institutions with insignificant transport needs.

**Table 2:** Inventory for the transportation of FW generated to SWDS

**Institution**	${\varvec{y}}$**(km)**	${{\varvec{P}}}_{{\varvec{A}}{\varvec{x}}}$**(kg/**day**)**	**W (tkm/annum)**
1	13.2	101.95	491.20
2	5.5	113.44	227.73
3	-	51.08	-

## Results and discussion

### FW generation statistics and management practices

#### FW generation statistics

The average daily FW generated (W_A_) from the operations at the hotels during the audit periods are provided in Table 3. The FW composition within the waste being generated at hospitality institutions confirms the predominant composition of FW in the waste generated within the hospitality sector. The need for sustainable FW management arises through the recovery of nutrients, materials, and energy from FW.

**Table 3:** Audit findings of the FW generation audit

Institution	${{\varvec{R}}0}_{{\varvec{a}}{\varvec{x}}}$(%)	${{\varvec{R}}{\varvec{O}}}_{{\varvec{A}}{\varvec{x}}}$(%)	FW Composition (%)	${{\varvec{P}}}_{{\varvec{a}}{\varvec{x}}}$(kg/day)	${{\varvec{P}}}_{{\varvec{A}}{\varvec{x}}}$(kg/day)	P_Ax_/room occupied
1	100.00%	56.00%	72.00	182.05	101.95	1.01
2	61.95%	47.58%	72.00	147.70	113.44	2.25
2	85.33%	39.00	64.00	111.76	51.08	1.62

Table 4 shows the comparison of the waste generation estimates for the respective hotels against reported estimates in other jurisdictions. The waste generation figures for the audited hotel institutions in Zimbabwe are well within the reported figures from other reports and jurisdictions of between 1.00 and 2.50 kg/day per room occupied/guest shown in Table 4. Although the waste generation figures are within the reported values in Table 2, they are lower than the 4 kg/room/day reported for a 4-star hotel in Hoi An, Vietnam (Hoang et al., 2017). The waste generation figures obtained during the assessment are generally within the ranges of 1.71, 2.32, and 6.57 kg/guest/day reported by Son et al. (2018) for three, four, and five-star hotels, respectively, in Hue City, Vietnam. Maximum values of 3.33 kg/guest/day were also reported in Asia (Chan & Lam, 2001; Omidiani & Hashemihezaveh, 2016).

**Table 4:** Comparison of the waste generation figures for the respective hotels against reported figures in other jurisdictions

Hotel	Estimated waste generation (kg/day per room occupied/guest)	Reported figures from other sources (kg/day/per room occupied/guest)			
		UNEP 2003		(Bjørn Olsen et al., 2018)	(Pham Phu et al., 2018)
1	1.01	1.00*	2.00**	1.60	2.50***
2	2.25				
3	1.62				

*European tourists

** American tourists

*** In Vietnam, 58.5% of waste was biodegradable

#### FW management practices

Table 5 provides the FW management information regarding separation, collection, treatment, and disposal methods at the respective institutions. Source separation of FW is currently being practiced at 1st and 3rd percentiles, especially within kitchens and dining rooms. However, the source-separated FW at site 1 is sent to a waste collection point where municipal waste collection trucks collect the waste indiscriminately by mixing the source-separated FW with other waste fractions for final disposal at the landfill or dumpsite. This renders FW source separation a futile exercise, hence the need for an offtake system in the form of composting or anaerobic digestion (AD) for the FW. At site 2, FW is indiscriminately collected by municipal waste collection trucks for final disposal at the dumpsite. At 3, the FW is source separated and subjected to partial composting that was regarded the same as disposal in a shallow SWDS.

**Table 5:** FW management practices at the audited institutions

Institution	Source separation	Indiscriminate collection	Dumpsite	Composting
1	√	√	√	
2		√	√	
3	√		√	√

### IPCC guidelines based on GHG emission estimates

**Fig. 4:**
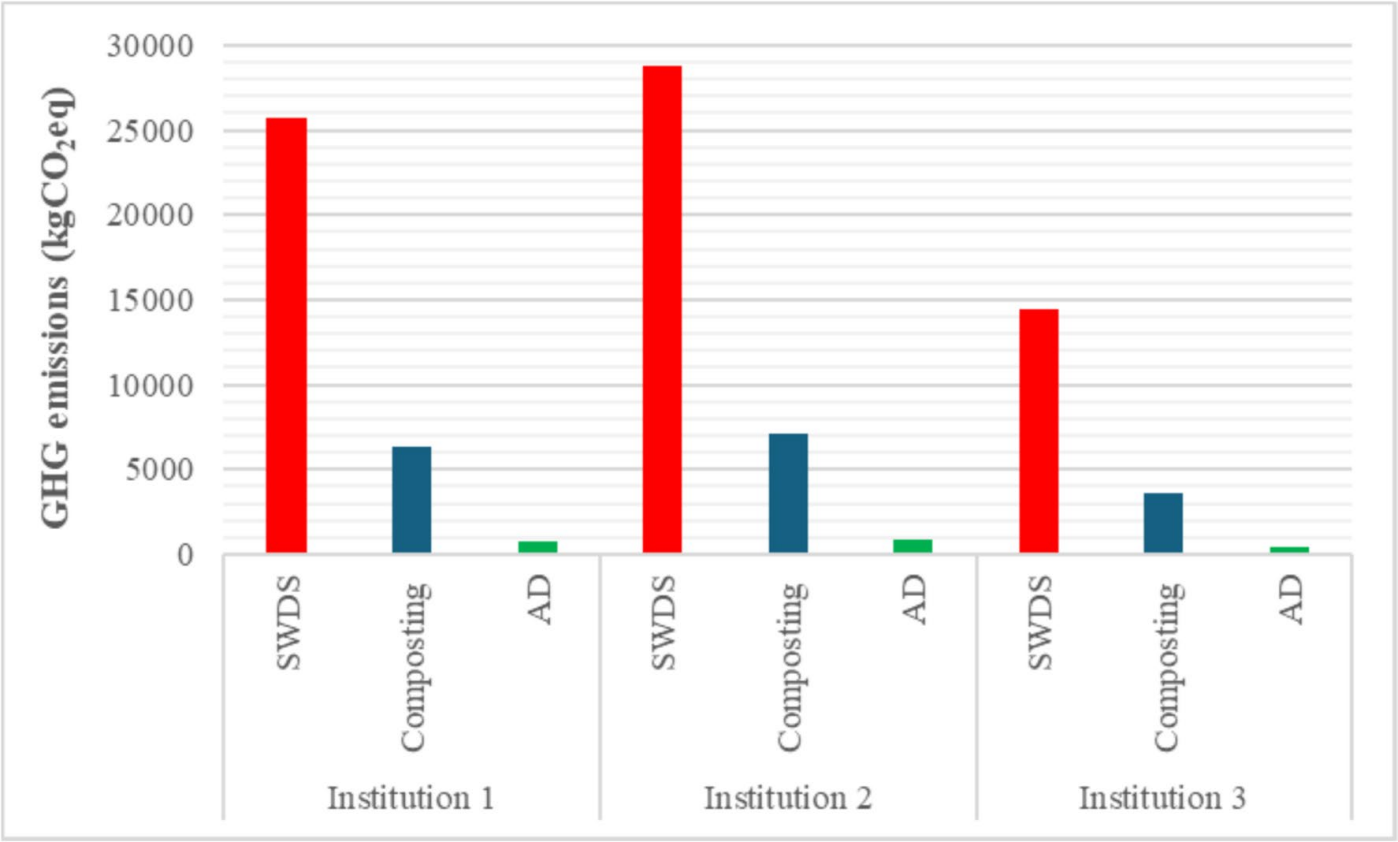
Graphical illustrations of the comparisons of the GHG emissions from dumping, composting, and AD

The GHG emissions from the disposal of FW generated at SWDS, during proposed composting and AD, were estimated using the IPCC guidelines and are given in Table 6. Figure 4 provides graphical illustrations of the comparisons of GHG emissions from dumping, composting, and AD of the FW generated at the hotels. The GHG emissions from the disposal of FW at SWDS were estimated at 6.90 × 10^2^ kgCO_2_eq per ton of FW being the highest. Composting with GHG emissions estimated at 1.71 × 10^2^ kgCO_2_eq per ton of FW follows indicating lower GHG emissions than FW disposal at SWDS, which is currently practiced. Composting thus results in a 75% reduction in GHG emissions. AD has the lowest GHG emissions estimated at 2.00 × 10^1^ kgCO_2_eq per ton of FW that leads to a maximum reduction in GHG emissions of 97% when compared to the disposal of FW at SWDS. The reported reduction in GHG emissions associated with the AD and composting of FW confirms the conclusions by Lunag and Elauria (2021) in their literature review that composting and AD are practical, appropriate, and sustainable biowaste management methods. The reductions in GHG emissions associated with AD show its environmental friendliness. Lin et al. (2022) reported 161% environmental impact reductions associated with the generation of electricity using biogas derived from the AD of FW when compared to open landfilling or dumping of the FW.

**Table 6:** Estimates of GHG emissions from disposal at solid waste disposal sites (dumpsites), composting, and AD of FW generated at selected hotels

Institution	1			2			3		
**GHG**	**SWDS**	**Composting**	**AD**	**SWDS**	**Composting**	**AD**	**SWDS**	**Composting**	**AD**
CH_4_ (Gg)	1.03 × 10^−3^	1.95 × 10^−4^	2.98 × 10^−5^	1.15 × 10^−3^	1.67 × 10^4^	3.34 × 10^5^	5.79 × 10^−4^	8.40 × 10^−5^	1.68 × 10^−5^
N_2_O (Gg)	-	8.95 × 10^−6^	-	-	1.00 × 10^5^	-	-	5.04 × 10^−6^	0
Total GHG (kgCO_2_eq)	2.57 × 10^4^	6.39 × 10^3^	7.46 × 10^2^	2.88 × 10^4^	7.15 × 10^3^	8.34 × 10^2^	1.45 × 10^4^	3.60 × 10^3^	4.20 × 10^2^
Total GHG (kgCO_2_eq/ton FW)	6.90 × 10^2^	1.71 × 10^2^	2.00 × 10^1^	6.90 × 10^2^	1.71 × 10^2^	2.00 × 10^1^	6.90 × 10^2^	1.71 × 10^2^	2.00 × 10^1^
% Reduction	-	75%	97%	-	68%	96%	-	75%	97%

### ELCA

The ELCA results reveal the impacts of disposal at SWDS, composting, and AD of FW across several environmental impact categories, unlike the IPCC guidelines, which specifically focus only on global warming impacts. Individual ELCA studies on FW have focused on specific impact categories, as indicated in the studies reviewed by Batool et al. (2024). The results presented herein are for all the impact categories under SimaPro shown in Table 7.

**Table 7:** SimaPro impact categories

Impact category	Unit
Global warming potential (GW)	kg CO_2_ eq
Stratospheric ozone depletion (SOD)	kg CFC11 eq
Ionizing radiation (IO)	kBq Co60 eq
Ozone formation, human health (OF-HH)	kg NOx eq
Fine particulate matter formation	kg PM2.5 eq
Ozone formation, terrestrial ecosystems (OF-TE)	kg NOx eq
Terrestrial acidification (TA)	kg SO_2_ eq
Freshwater eutrophication	kg P eq
Marine eutrophication	kg N eq
Terrestrial ecotoxicity (TE)	kg 1,4-DCB
Freshwater ecotoxicity	kg 1,4-DCB
Marine ecotoxicity	kg 1,4-DCB
Human carcinogenic toxicity (HCT)	kg 1,4-DCB
Human noncarcinogenic toxicity (HNCT)	kg 1,4-DCB
Land use (LU)	m^2^a crop eq
Mineral resource scarcity (MRS)	kg Cu eq
Fossil resource scarcity (FRS)	kg oil eq
Water consumption (WC)	m^3^

#### Global warming

**Fig. 5:**
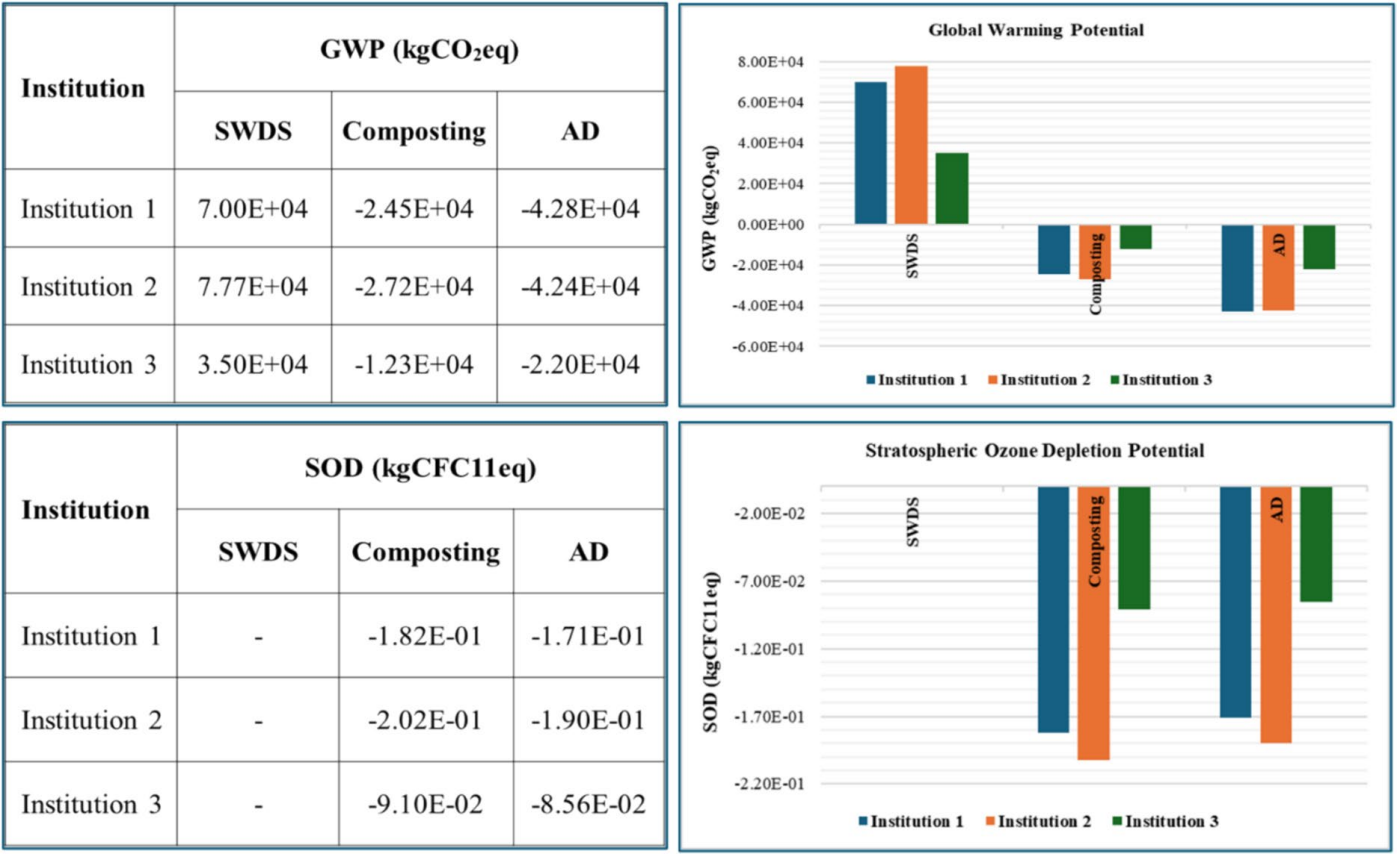
Effects of GWP and SODP disposal at SWDS and composting and AD of FW generated at the selected institutions

The disposal of FW in SWDS results in a positive GW impact with the emission of 1.88 × 10^3^ kgCO_2_eq per ton of FW. The GWP impacts of dumping FW in SWDS are shown in Fig. 5. These findings confirm those of other ELCA studies reported by Fu et al. (2021) and Kurniawan et al. (2023) that the disposal of FW in SWDS, including landfills, results in the greatest net positive GW impact. Batool et al. (2024) ranked the different FW management and treatment technologies based on findings from ELCA studies, with the disposal of FW in SDWSs having a major impact on the environment. The GWP results from the emission of methane (CH_4_), which is generated from the anaerobic decomposition of FW in SWDS. The potential recovery and use of landfill CH_4_ can significantly reduce the impact of GW.

Both composting and AD had positive impacts on GWP, albeit the impact was lower than that of FW disposal in SWDS. The AD process contributes to 2.92 × 10^2^ kgCO_2_eq per ton of FW. Composting contributes to 6.04 × 10^1^ kgCO_2_eq per ton of FW. When the avoided GHG emissions from the production of NPK (nitrogen (N), phosphorous (P), and potassium (K)) fertilizers estimated at 7.18 × 10^2^ kgCO_2_eq per ton of FW used in the production of compost to replace NPK fertilizers, composting will have a net negative impact on GW, as shown in Fig. 5. The renewable energy production capability of AD gives it an edge over composting. Overall composting brings about a net GWP of 6.58 × 10^2^ kgCO_2_eq per ton of FW used in the production of compost to replace NPK fertilizers. The superior performance of AD was also observed by Woon et al. (2021) who reported electricity generation from the AD of FW as the best environmentally friendly scenario associated with the avoidance of human health and ecosystem quality impacts in the magnitude of 4.92 × 10^−4^ DALY and 6.30 species.yr. Woon et al. (2021) further noted that utilization of 80% of the FW generated in Malaysia to generate electricity results in a 0.4% reduction of total carbon emissions and a 1.1% contribution to the entire national electricity mix. Lin et al. (2022) reported 146% human health and 161% environmental impact reductions that come with the generation of electricity using biogas derived from the AD of FW when compared to open landfilling or dumping of the FW.

Despite its higher GWP per ton of FW before factoring in the avoided emissions from the production of NKP fertilizers which are replaced by compost or organic fertilizer from the AD digestate as well as the energy replaced by biogas produced during AD, AD results in a greater net negative GWP impact than composting, as shown in Fig. 5. Overall AD was estimated to bring about a net negative GWP of 1.15 × 10^3^, 1.02 × 10^3^, and 1.183 × 10^3^ kgCO_2_eq per ton of FW at institutions 1, 2, and 3, respectively. The difference in the net negative GWP for AD is due to the different energy use configurations considered during the assessment. A net negative GWP impact indicates a net environmental benefit. These findings confirm those of Nyitrai et al. (2023), who reported that the AD of FW leads to a net improvement in GWP. AD is therefore more beneficial to the environment or preferable to composting regarding GWP. Xu et al. (2015) considered AD among the best FW management options, and Eriksson et al. (2015) observed the comparative benefits of AD over composting.

#### Stratospheric ozone depletion

The disposal of FW in SWDS does not contribute to SOD activity. The composting and AD of FW contribute 2.22 × 10^−4^ and 6.21 × 10^−4^kgCFC11eq. When the avoided emissions are considered, composting and AD resultantly have net negative SOD potentials of − 4.88 × 10^−3^ and − 4.59 × 10^−3^kgCFC11eq. The net negative SOD shows a net positive environmental benefit from composting and AD. The results are shown in Fig. 5. Batool et al. (2024) observed and reported that the AD of FW is the best-performing technology for SOD activity, i.e., it performs better than both the composting of FW and the disposal of FW in SWDS. This study’s findings disagree with the findings of Batool et al. (2024), who indicated that composting performs poorly compared to disposal at SWDS. This discrepancy could be attributed to landfilling being the SWDS considered in the review by Batool et al. (2024), whereas this study considered open dumping in shallow unsanitary landfills with no landfill gas capture or recovery as well as landfill leachate treatment.

#### Ozone formation potential

**Fig. 6:**
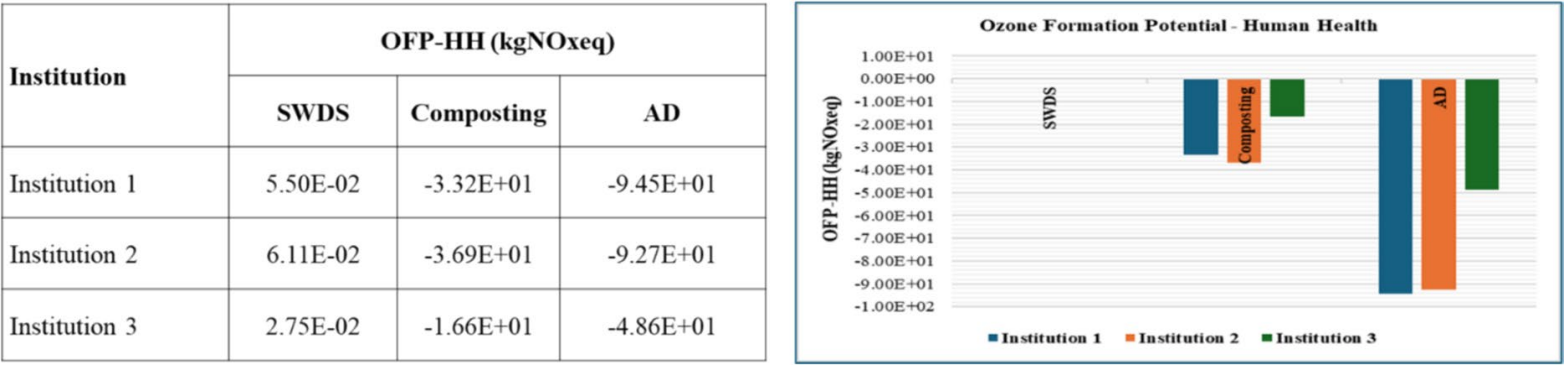
OZ impacts of disposal at SWDS, composting, and AD of FW generated at the selected institutions

The ozone formation potential (OFP) was assessed regarding human health and terrestrial ecosystems. The results revealed the same magnitude of impact on both human health and terrestrial ecosystems based on OFP. The results revealed slight variations, with terrestrial ecosystems exhibiting 2% greater variability than the estimates for human health. Figure 6 shows the results regarding human health-based OFP. The disposal of FW in SWDS leads to positive OFPs estimated at 1.48 × 10^3^ kg NOxeq per ton of FW for human health- and terrestrial ecosystem-based OFPs. As a result, there was a net positive OFP for both human health and terrestrial ecosystems based on an OFP of 2.95 × 10^3^ kg NOxeq per ton of FW disposed at an open dumpsite.

Composting and AD of FW produce volatile organic compounds (VOCs) (Cui et al., 2022; Mustafa et al., 2017; Nie et al., 2018, 2019), which were regarded by Shao et al. (2011) and Gong et al. (2017) as the main precursors to ozone formation. The study results showed that composting results in human health- and terrestrial ecosystem-based OFP of 1.63 × 10^−1^ and 1.69 × 10^−1^ kg of NOxeq per ton of FW, respectively. Therefore, composting contributes 3.32 × 10^−1^ kg of NOxeq per ton of FW to both human health and terrestrial ecosystem-based OFP. Regarding AD, it contributes to human health and terrestrial ecosystems OFP of 2.81 × 10^−1^ and 2.84 × 10^−1^ kg of NOxeq per ton of FW, respectively. Therefore, AD contributes 5.65 × 10^−1^ kg of NOxeq per ton of FW to both human health and terrestrial ecosystem-based OFPs. However, when the avoided emissions through the replacement of NPK fertilizers or renewable energy, composting and AD result in net negative OFPs of − 1.78 × 10^0^ and − 4.48 × 10^0^ kgNOxeq per ton of FW for both human health and terrestrial ecosystem-based OFPs.

#### Ionizing radiation potential

**Fig. 7:**
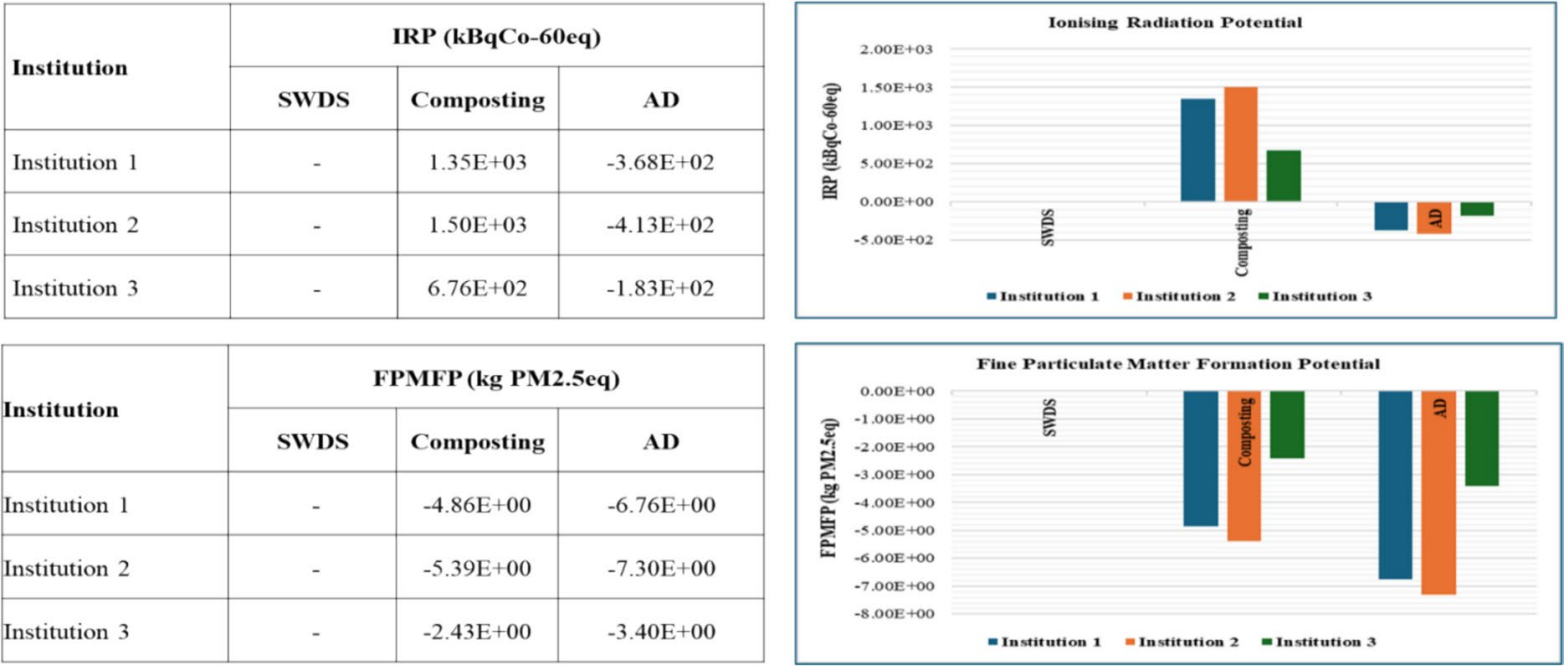
IR and FPMF impacts on disposal at SWD, composting, and AD of FW generated at the selected institutions

The results showed that the disposal of FW at SWDS has no impact on IRP, whereas composting has an IRP of 4.55 × 10^1^ kBqCo-60 eq per ton of FW. This means composting of FW has a net positive IRP; thus, it brings about negative impacts even after considering the avoided emissions from the replacement of chemical fertilizers with compost. Although the AD process has a positive IR of 8.35 × 10^−1^ kBqCo-60 eq/ton of FW, when the avoided emissions from the replacement of LPG gas and grid electricity with AD-derived biogas as well as the replacement of chemical fertilizers with AD-derived compost are considered, the AD system has negative IRP of 9.98 × 10^0^ kBqCo-60 eq/ton of FW; hence, it brings about environmental benefits. The IRP from the disposal of FW at SWDS, composting, and AD are shown in Fig. 7.

#### Fine particulate matter formation potential

The disposal of FW at SWDS leads to no FPMFP. The AD and composting of FW were projected to generate 2.46 × 10^−2^ and 3.00 × 10^−2^ kg PM2.5 eq per ton of FW, respectively. These findings showed that AD and the composting of FW have positive FPMFP. However, when avoided emissions that come with the replacement of chemical fertilizers by compost or organic fertilizer from composting and AD as well as from the replacement of LPG and grid electricity by AD-derived biogas are considered, both composting and AD systems result in net negative FPMFP. These results are shown in Fig. 7.

#### Terrestrial acidification potential

**Fig. 8:**
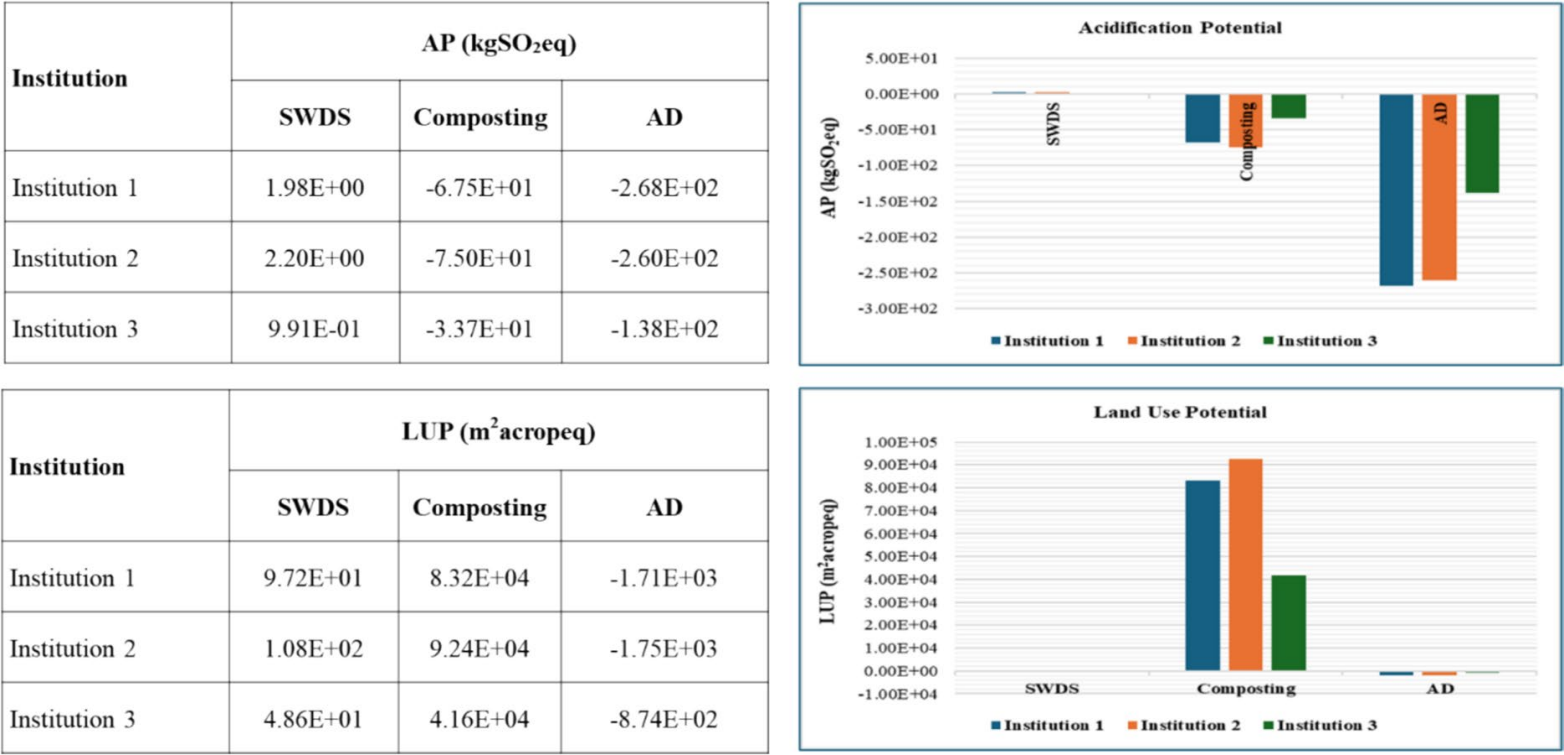
TA and LU impacts of disposal at SWDS, composting, and AD of FW generated at the selected institutions

The disposal of FW at SWDS, composting, and AD of FW lead to positive terrestrial acidification potential (TAP) impacts of 5.32 × 10^−2^, 5.42 × 10^−1^, and 2.94 × 10^−1^ kgSO2eq per ton of FW, respectively. The results showed that AD has the least TAP, confirming the findings of Batool et al. (2024), who identified TAP among the high LCA impacts from the disposal of FW at SWDS and the treatment of FW through composting. The avoided emissions from the replacement of chemical fertilizers by compost or organic fertilizer from composting and AD as well as the replacement of LPG and grid electricity by AD-derived biogas resulted in net negative TAP for both composting and AD systems, as shown in Fig. 8.

#### Land use potential

The disposal of FW at SWDS and the treatment of FW through AD lead to land use potential (LUP) of 2.61 × 10^0^ and 2.53 × 10^0^ m^2^acropeq per ton of FW, respectively. Composting had the highest LUP of 2.26 × 10^3^ m^2^acropeq per ton of FW. These findings also support those reviewed by Batool et al. (2024), who identified LU among the high LCA impact categories for the treatment of FW through composting. Factoring in the avoided emissions from the replacement of chemical fertilizers with organic fertilizers as well as LPG and grid electricity with biogas leads to overall negative LUP for the AD system. LU results for the disposal of FW at SWDS, composting, and AD are shown in Fig. 8.

#### Freshwater and marine eutrophication

**Fig. 9:**
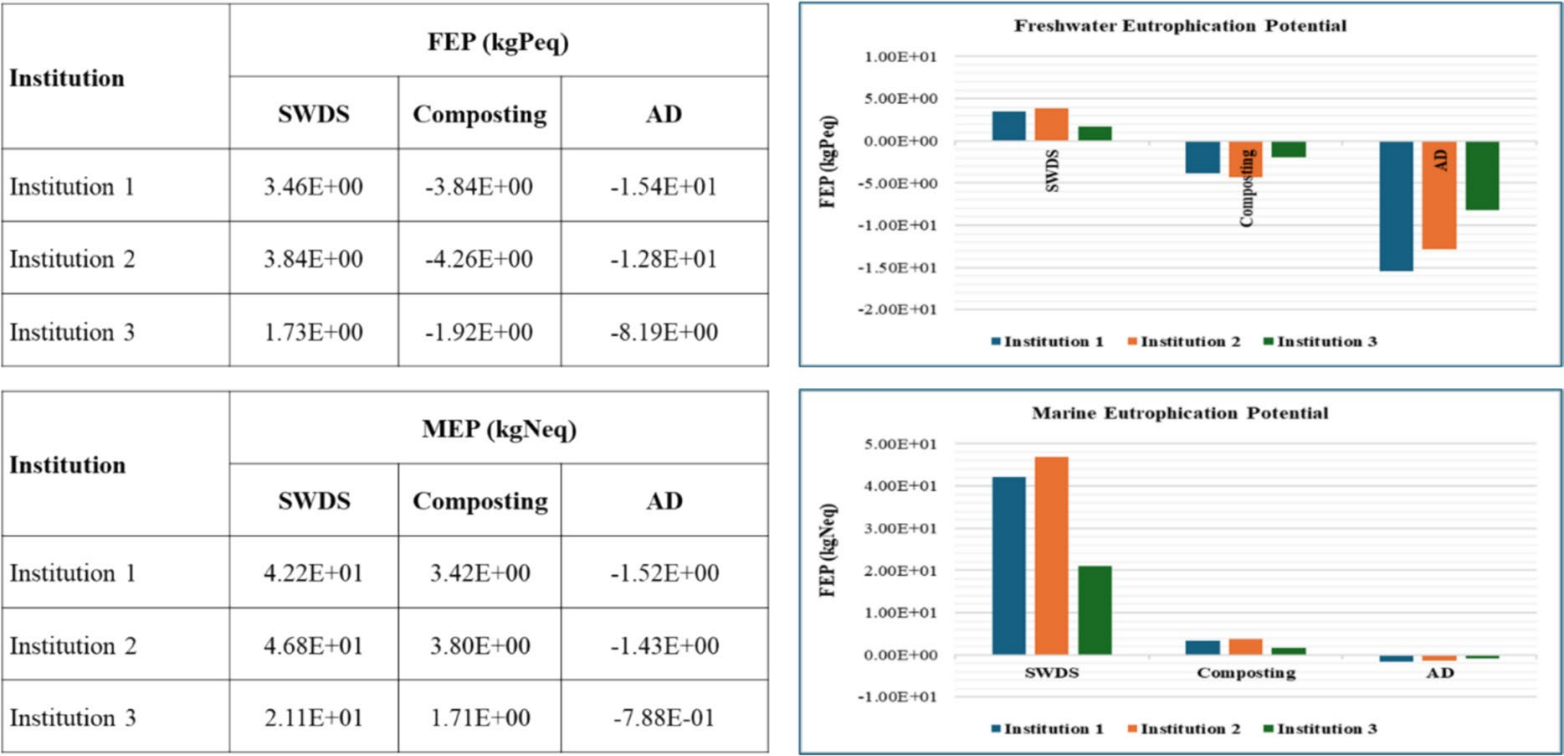
FEP and MEP of disposal at SWDS, composting, and AD of FW generated at the selected institutions

The freshwater eutrophication potential (FEP) and marine eutrophication potential (MEP) impact assessment results are provided in Fig. 9. The disposal of FW at SWDS, composting, and AD of FW were estimated to contribute to positive FEP of 9.27 × 10^−2^, 2.06 × 10^−2^, and 2.52 × 10^−1^ kgPeq per ton of FW, respectively. AD has the highest FEP, followed by disposal at SWDS. Regarding MEP, disposal at SWDS, composting, and AD was estimated to contribute 1.13 × 10^−1^, 1.14 × 10^−1^, and 1.45 × 10^−2^ kgNeq per ton of FW, respectively. Avoided emissions from the replacement of chemical fertilizers with compost or biofertilizer from the composting of FW result in net negative emissions with regards to FEP. Net positive MEP was estimated even after considering avoided emissions from the replacement of chemical fertilizers with compost derived from the composting of FW. On the contrary, AD had both net negative FEP and MEP when avoided emissions from the replacement of chemical fertilizers with AD-derived organic fertilizer together with the replacement of LPG and grid electricity with biogas derived from the AD system.

#### Terrestrial, freshwater, and marine ecotoxicity

**Fig. 10:**
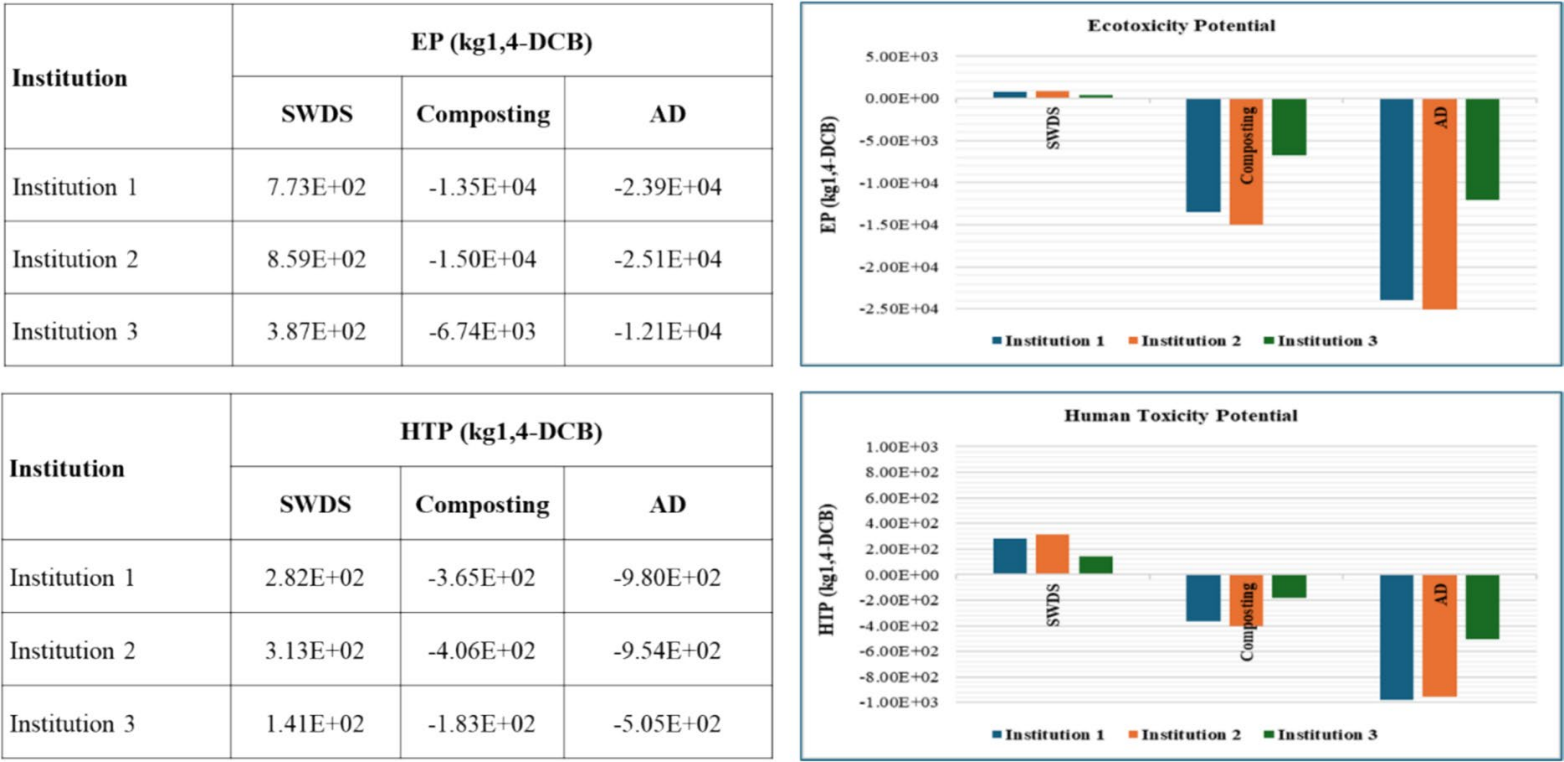
Ecotoxicity and human toxicity potentials at SWDS disposal, composting, and AD of FW generated at the selected institutions

The ecotoxicity potential (EP) consisting of terrestrial ecotoxicity potential (TEP) freshwater ecotoxicity potential and marine ecotoxicity potential assessment results are provided in Fig. 10. The disposal of FW at SWDS, composting, and AD of FW were estimated to contribute to positive TEPs of 5.42 × 10^−1^, 1.07 × 10^2^, and 5.06 × 10^1^ kg1,4-DCB per ton of FW, respectively. Likewise, positive freshwater ecotoxicities for the disposal of FW at SWDS, composting, and AD of FW of 1.53 × 10^1^, 4.9 × 10^0^, and 2.27 × 10^0^ kg1,4-DCB per ton of FW, respectively, were observed. Positive marine ecotoxicities were also estimated for the disposal of FW at SWDS, composting, and AD of FWof 4.88 × 10^0^, 1.51 × 10^0^, and 6.65 × 10^0^ kg1,4-DCB per ton of FW, respectively. When the avoided emissions from the use of compost or organic fertilizer and biogas are considered, both composting and AD systems result in net negative terrestrial, freshwater, and marine ecotoxicity, as shown in Fig. 10, with AD having the highest negative ecotoxicity, indicating the greatest environmental benefit.

#### Human toxicity potential

The human toxicity potential (HTP) was assessed based on the human carcinogenic toxicity potential (HCTP) and human noncarcinogenic toxicity potential (HNCTP) whose results are shown in Fig. 10. The disposal of FW at SWDS, composting, and AD of FW was estimated to contribute to positive HCTPs of 4.18 × 10^−2^, 9.01 × 10^−2^, and 6.91 × 10^−2^ kg 1,4-DCB per ton of FW, respectively. Likewise, positive HNCTPs were observed for the disposal of FW at SWDS, composting, and AD of FW of 7.52 × 10^0^, 3.06 × 10^0^, and 4.56 × 10^0^ kg1,4-DCB per ton of FW, respectively. Just as the case with EP, when the avoided emissions from the use of compost or organic fertilizer and biogas are considered, both composting and AD systems result in net negative HTP (HCTP and HNCTP), as shown in Fig. 10, with AD having the highest negative HTP, indicating the greatest environmental and human health benefit. The inventory for the AD process included the post AD treatment or stabilization of the AD digestate. This is regarded vital to reduce the HTP impacts from its direct application noted by Arias et al. (2021).

#### Mineral and fossil resource scarcity

**Fig. 11:**
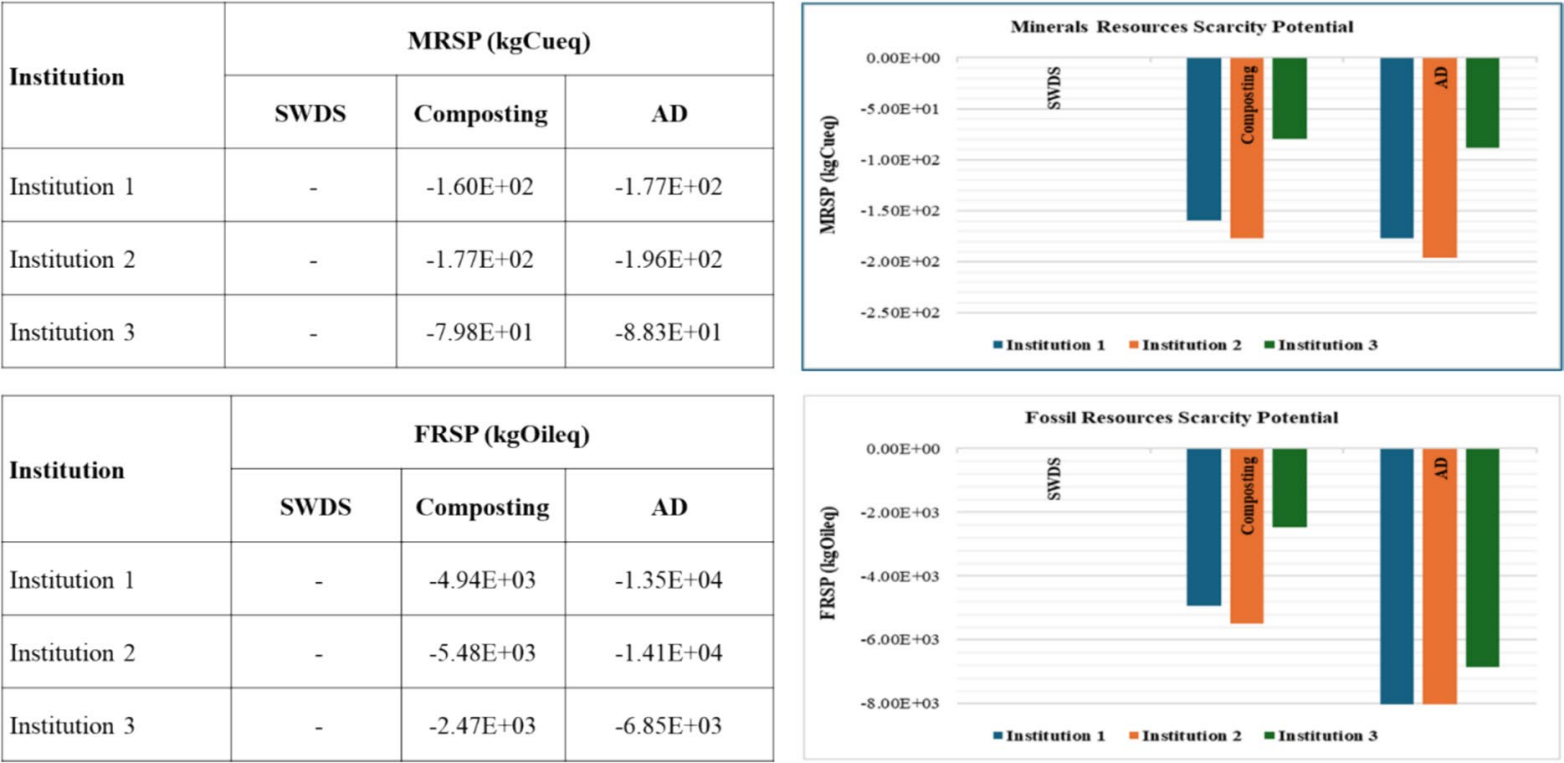
MRSP and FRSP of disposal at SWDS, composting, and AD of FW generated at the selected institutions

The results for mineral resource scarcity potential (MRSP) and fossil resource scarcity potential (FRS P) are shown in Fig. 11. The disposal of FW at SWDS does not contribute to MRSP and FRSP. The composting and AD of FW were estimated to contribute to positive MRSPs of 4.68 × 10^−1^ and 1.21 × 10^−1^ kgCueq per ton of FW, respectively. Likewise, positive FRSPs were observed for the composting and AD of FW estimated at 8.83 × 10^0^ and 1.35 × 10^1^ kgoileq per ton of FW, respectively. The avoided emissions from the use of compost or organic fertilizer and biogas for the composting and AD systems led to net negative MRSPs and FRSPs, as shown in Fig. 11, with AD also having the highest net negative MRSP and FRSP, indicating the greatest environmental benefit.

#### Water consumption potential

**Fig. 12:**
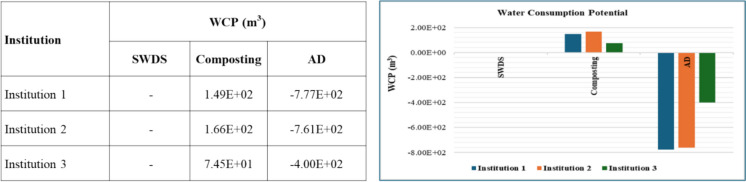
WCPs of disposal at SWDS, composting, and ADs of FW generated at the selected institutions

The results for water consumption potential (WCP) are shown in Fig. 12. No WCP impacts from the disposal of FW at SWDS. The composting and AD of FW were estimated to contribute to positive WCPs of 1.13 × 10^1^ and 2.10 × 10^−1^ m^3^ per ton of FW, respectively. However, even after considering the avoided emissions from the use of compost or organic fertilizer instead of chemical fertilizers, the composting of FW has a net positive effect on WCP of 4 m^3^ per ton of FW. AD systems led to a net negative WCP after considering the avoided emissions from the use of compost or organic fertilizer and biogas, as shown in Fig. 12.

#### Sensitivity analysis results

The sensitivity analysis results have shown that the transportation of FW to SWDS will lead to increased impacts across all the impact categories. Table 8 shows the sensitivity analysis results for the management of FW generated at institution through the disposal at SWDS. The increases in transportation-related impacts are lower for institution 2, further confirming the system sensitivity since institution 2 has lower tkm of FW compared to institution 1.

**Table 8:** Sensitivity analysis results factoring transportation of FW to SWDS-related impacts

Impact category	Transportation	Disposal at SWDS	Impact/ton of FW
Global warming (kg CO2 eq)	639.17	69955.02	1893.62
Stratospheric ozone depletion (kg CFC11 eq)	0.00	0.00	0.00
Ionizing radiation (kBq Co-60 eq)	4.12	0.00	0.11
Ozone formation, human health (kg NOx eq)	4.44	0.06	0.12
Fine particulate matter formation (kg PM2.5 eq)	0.35	0.00	0.01
Ozone formation, terrestrial ecosystems (kg NOx eq)	4.65	0.06	0.13
Terrestrial acidification (kg SO2 eq)	2.19	1.98	0.11
Freshwater eutrophication (kg P eq)	0.01	3.46	0.09
Marine eutrophication (kg N eq)	0.00	42.17	1.13
Terrestrial ecotoxicity (kg 1,4-DCB)	375.00	20.20	10.60
Freshwater ecotoxicity (kg 1,4-DCB)	2.84	571.01	15.39
Marine ecotoxicity (kg 1,4-DCB)	1.07	181.96	4.91
Human carcinogenic toxicity (kg 1,4-DCB)	0.08	1.56	0.04
Human non-carcinogenic toxicity (kg 1,4-DCB)	2.53	280.40	7.59
Land use (m2a crop eq)	4.07	97.19	2.72
Mineral resource scarcity (kg Cu eq)	0.26	0.00	0.01
Fossil resource scarcity (kg oil eq)	197.26	0.00	5.29
Water consumption (m3)	0.31	0.00	0.01

#### Ranking of the FW management and treatment methods

**Fig. 13:**
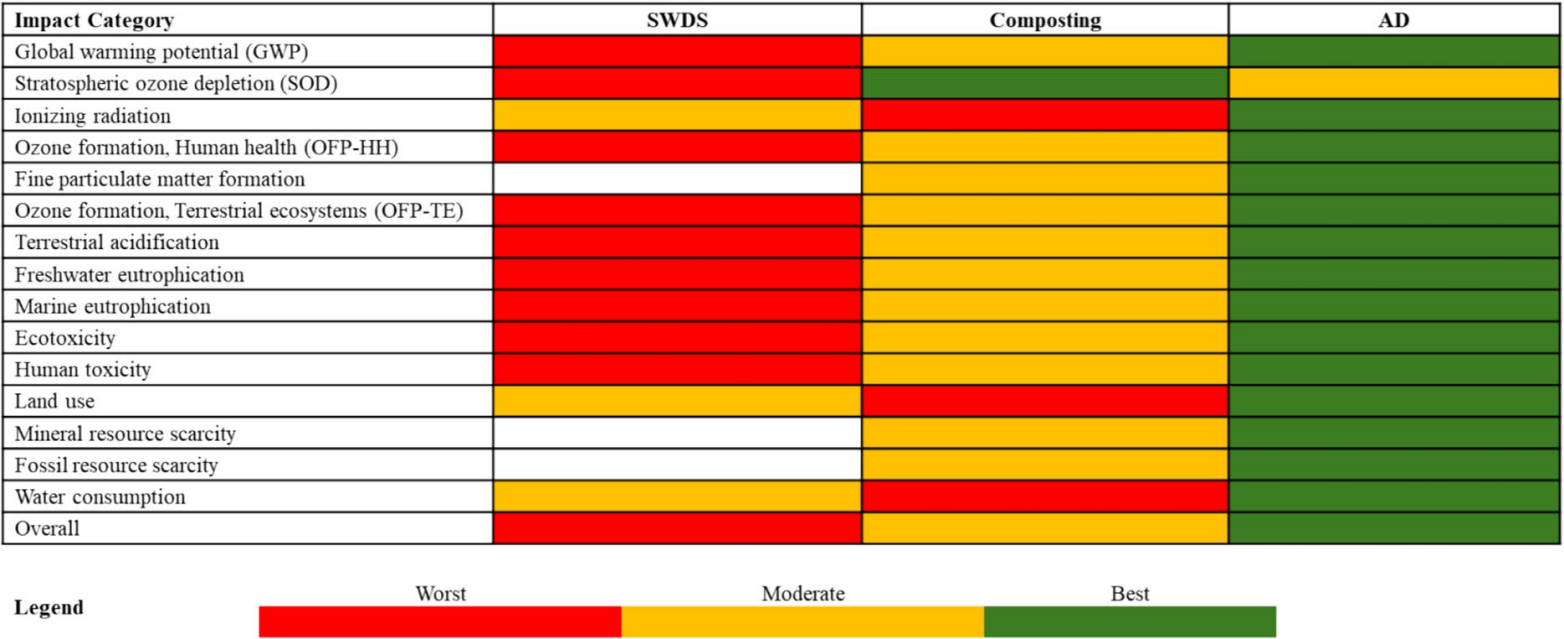
Ranking of the FW management and treatment methods

The impacts of the disposal of FW at SWDS, composting, and AD of FW were assessed and ranked based on their environmental performance against each of the life cycle impact categories. The ranking results are shown in Fig. 13, which provides a clearer picture of the environmental performance of the FW management and treatment methods. The best and worst environmental performance methods are assigned to each impact category, with the disposal of FW in SWDS being the worst method for most of the impact categories, namely, global warming, stratospheric ozone depletion, ozone formation-human health, ozone formation-terrestrial ecosystems, terrestrial acidification, freshwater eutrophication, marine eutrophication, ecotoxicity, and human toxicity. Composting was the worst for three impact categories, namely, ionizing radiation, land use, and water consumption. AD was the best-performing method across all the impact categories for stratospheric ozone depletion, after which it had the second-highest occurrence after composting. Therefore, overall, AD is the best method for determining net negative environmental impacts, which is consistent with the findings of Fu et al. (2023) and Batool et al. (2024). The disposal of FW at SWDS is thus the worst FW management and treatment method.

## Conclusion

The average FW generation within the Zimbabwean hospitality industry was estimated to be 1.63 kg/guest/day, with a maximum and minimum of 1.01 and 2.25 kg/guest/day, respectively. These waste generation figures are lower than those reported in other studies and from other jurisdictions. Interestingly, source separation of FW is currently being practiced, although the source-separated FW is indiscriminately collected by municipal waste collection trucks for final disposal at official landfills or dumpsites. This renders FW source separation a futile exercise, hence the need for an offtake system in the form of composting or anaerobic digestion (AD) for FW.

The GHG emissions from the disposal of FW generated at dumpsites estimated using the IPCC guidelines show that the disposal of FW at SWDS results in the highest GHG emissions at 6.90 × 10^2^ kgCO_2_eq per ton of FW. Composting with GHG emissions estimated at 1.71 × 10^2^ kgCO_2_eq per ton of FW follows indicating lower GHG emissions than FW disposal at dumpsites, which is currently practiced. Composting thus results in a maximum 75% reduction in GHG emissions. AD has the lowest GHG emissions estimated at 2.00 × 10^1^ kgCO_2_eq per ton of FW that leads to a maximum reduction in GHG emissions of 97% when compared to the disposal of FW at SWDS.

The results of the environmental life cycle assessment also showed that AD is the best method for leading to net negative environmental impacts, whereas the disposal of FW at SWDS was thus the worst FW management and treatment method. Therefore, steps need to be taken to ensure that either the composting or AD of FW is generated within the hospitality industry in Zimbabwe. These steps should also include addressing major policy deficiencies in Zimbabwe with regard to the absence of mandatory source separation of FW and prohibition of its disposal at SWDS. Policies aimed at enforcing the establishment of institutional food waste AD or composting facilities by large-scale FW generators such as the hospitality industries need to be enacted. These policies should also enhance the establishment of viable markets for FW which will promote the source separation of FW in the country. Further, the policy should provide for deterrence fines for FW generators who would have been found disposing of their FW illegally and in undesignated areas. Despite these policy deficiencies, Zimbabwe has its National Integrated Solid Waste Management Plan that came into effect in July 2014 which provides for the establishment of composting and AD facilities as well as source separation of biodegradable waste fractions including FW. The implementation of the plan proposals has largely been stagnant. The absence of policy on issues related to the plan proposals could be attributed to the non-implementation of the plan proposals. The plan is due for review, and one of the review recommendations should be the need for the promulgation of associated policies. This is envisaged to see the implementation of environmentally sustainable, economically viable, and socially acceptable FW management systems in Zimbabwe.

## Supplementary Information

Below is the link to the electronic supplementary material.

Supplementary file1 (ZIP 861 KB)

## Data Availability

No datasets were generated or analysed during the current study.
