# Supplementary material for: Inventory analysis and environmental life cycle impact assessment of hotel food waste management for bio-circular economy development in Zimbabwe
Source: Environ Monit Assess. 2024 Nov 14;196(12):1196. doi: 10.1007/s10661-024-13314-6 (PMC11564243; doi:10.1007/s10661-024-13314-6)
Supplement: Supplementary file 1 — Supplementary file1 (ZIP 861 KB) [file 10661_2024_13314_MOESM1_ESM.zip › GWP and SOD Figure.pptx]

## Slide 1
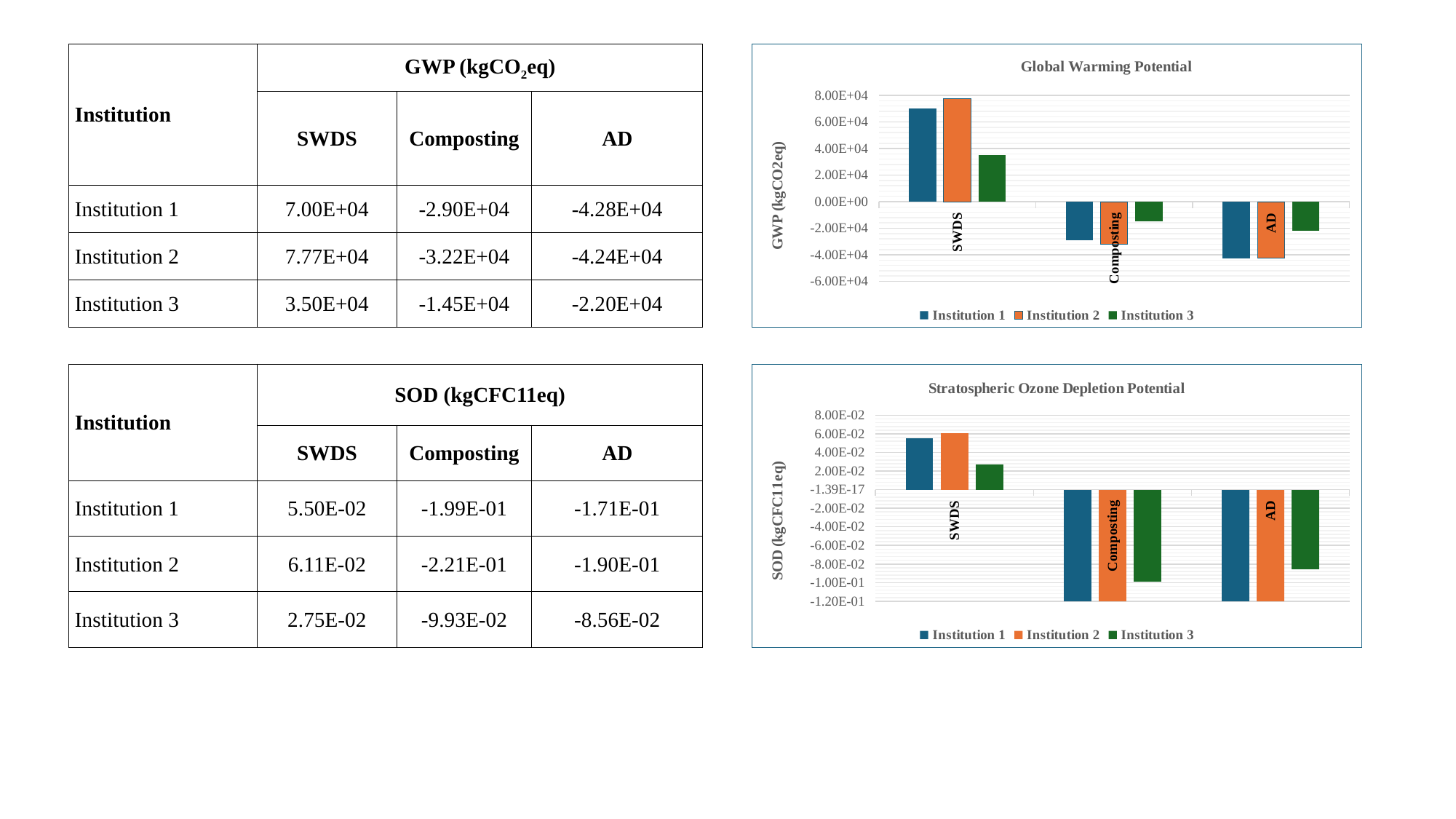

| Institution | GWP (kgCO2eq) | | |
| --- | --- | --- | --- |
| | SWDS | Composting | AD |
| Institution 1 | 7.00E+04 | -2.90E+04 | -4.28E+04 |
| Institution 2 | 7.77E+04 | -3.22E+04 | -4.24E+04 |
| Institution 3 | 3.50E+04 | -1.45E+04 | -2.20E+04 |
### Chart: Global Warming Potential
| Category | Institution 1 | Institution 2 | Institution 3 |
|---|---|---|---|
| SWDS | 69955.0235116482 | 77704.8691957444 | 34977.5117558241 |
| Composting | -29023.8110518737 | -32242.6192508763 | -14511.9055259368 |
| AD | -42849.8402228558 | -42364.0291059863 | -22001.2819117921 || Institution | SOD (kgCFC11eq) | | |
| --- | --- | --- | --- |
| | SWDS | Composting | AD |
| Institution 1 | 5.50E-02 | -1.99E-01 | -1.71E-01 |
| Institution 2 | 6.11E-02 | -2.21E-01 | -1.90E-01 |
| Institution 3 | 2.75E-02 | -9.93E-02 | -8.56E-02 |
### Chart: Stratospheric Ozone Depletion Potential
| Category | Institution 1 | Institution 2 | Institution 3 |
|---|---|---|---|
| SWDS | 0.0550439185280993 | 0.0611418633650374 | 0.0275219592640497 |
| Composting | -0.19853416248122 | -0.220552982469589 | -0.0992670812406098 |
| AD | -0.171107925353799 | -0.189882913054618 | -0.0855769037985113 |
