# Supplementary material for: Inventory analysis and environmental life cycle impact assessment of hotel food waste management for bio-circular economy development in Zimbabwe
Source: Environ Monit Assess. 2024 Nov 14;196(12):1196. doi: 10.1007/s10661-024-13314-6 (PMC11564243; doi:10.1007/s10661-024-13314-6)
Supplement: Supplementary file 1 — Supplementary file1 (ZIP 861 KB) [file 10661_2024_13314_MOESM1_ESM.zip › Institution 3 ELCA System Boundary.pptx]

## Slide 1
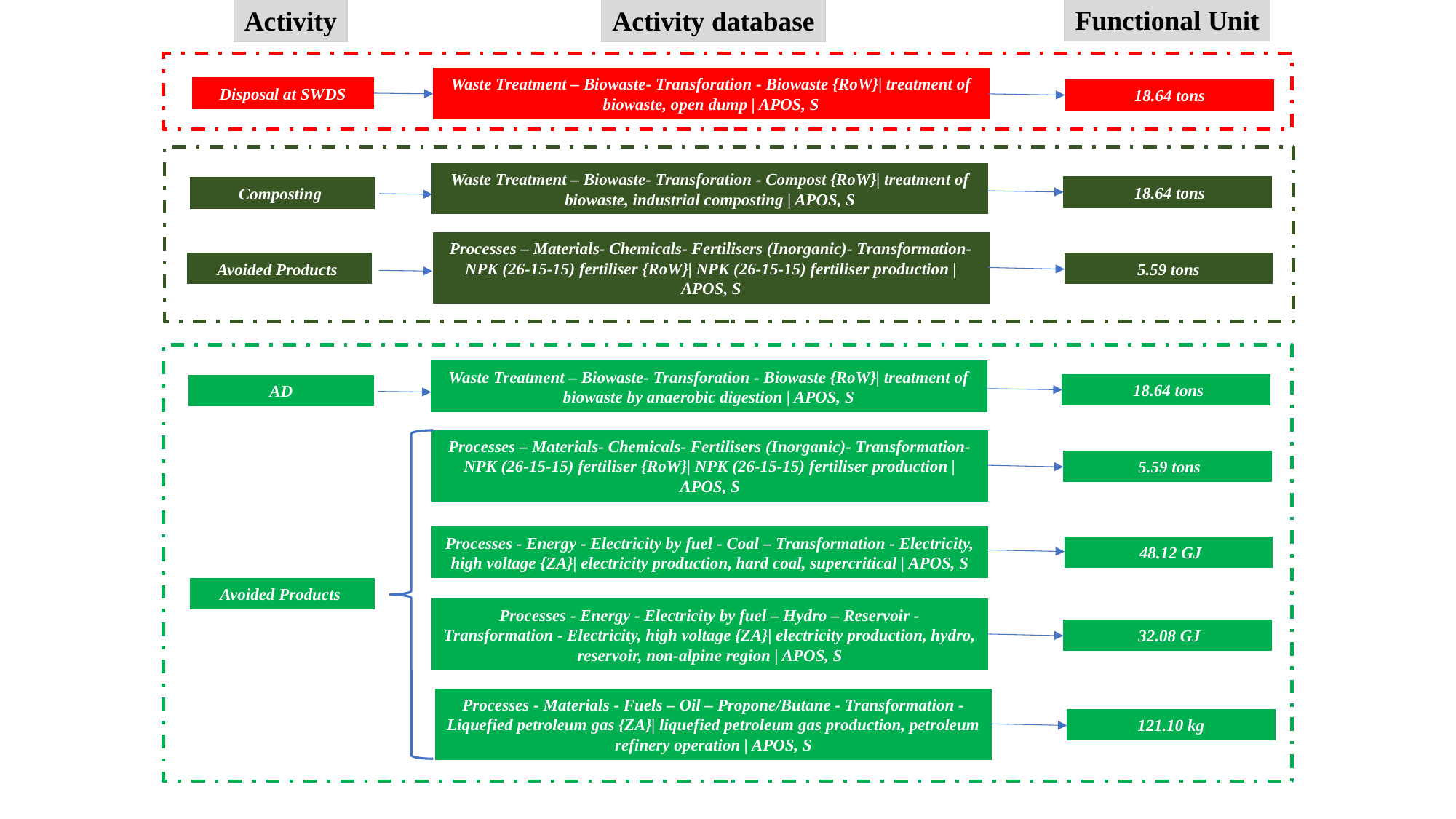

Functional Unit
Activity database
Activity
Waste Treatment – Biowaste- Transforation - Biowaste {RoW}| treatment of biowaste, open dump | APOS, S
Disposal at SWDS
18.64 tons
Waste Treatment – Biowaste- Transforation - Compost {RoW}| treatment of biowaste, industrial composting | APOS, S
 18.64 tons
Composting
Processes – Materials- Chemicals- Fertilisers (Inorganic)- Transformation-NPK (26-15-15) fertiliser {RoW}| NPK (26-15-15) fertiliser production | APOS, S
Avoided Products
5.59 tons
Waste Treatment – Biowaste- Transforation - Biowaste {RoW}| treatment of biowaste by anaerobic digestion | APOS, S
 18.64 tons
AD
Processes – Materials- Chemicals- Fertilisers (Inorganic)- Transformation-NPK (26-15-15) fertiliser {RoW}| NPK (26-15-15) fertiliser production | APOS, S
 5.59 tons
Processes - Energy - Electricity by fuel - Coal – Transformation - Electricity, high voltage {ZA}| electricity production, hard coal, supercritical | APOS, S
 48.12 GJ
Avoided Products
Processes - Energy - Electricity by fuel – Hydro – Reservoir - Transformation - Electricity, high voltage {ZA}| electricity production, hydro, reservoir, non-alpine region | APOS, S
 32.08 GJ
Processes - Materials - Fuels – Oil – Propone/Butane - Transformation - Liquefied petroleum gas {ZA}| liquefied petroleum gas production, petroleum refinery operation | APOS, S
121.10 kg
