# Supplementary figures and images for: Inventory analysis and environmental life cycle impact assessment of hotel food waste management for bio-circular economy development in Zimbabwe
Source: Environ Monit Assess. 2024 Nov 14;196(12):1196. doi: 10.1007/s10661-024-13314-6 (PMC11564243; doi:10.1007/s10661-024-13314-6)

## Slide 1
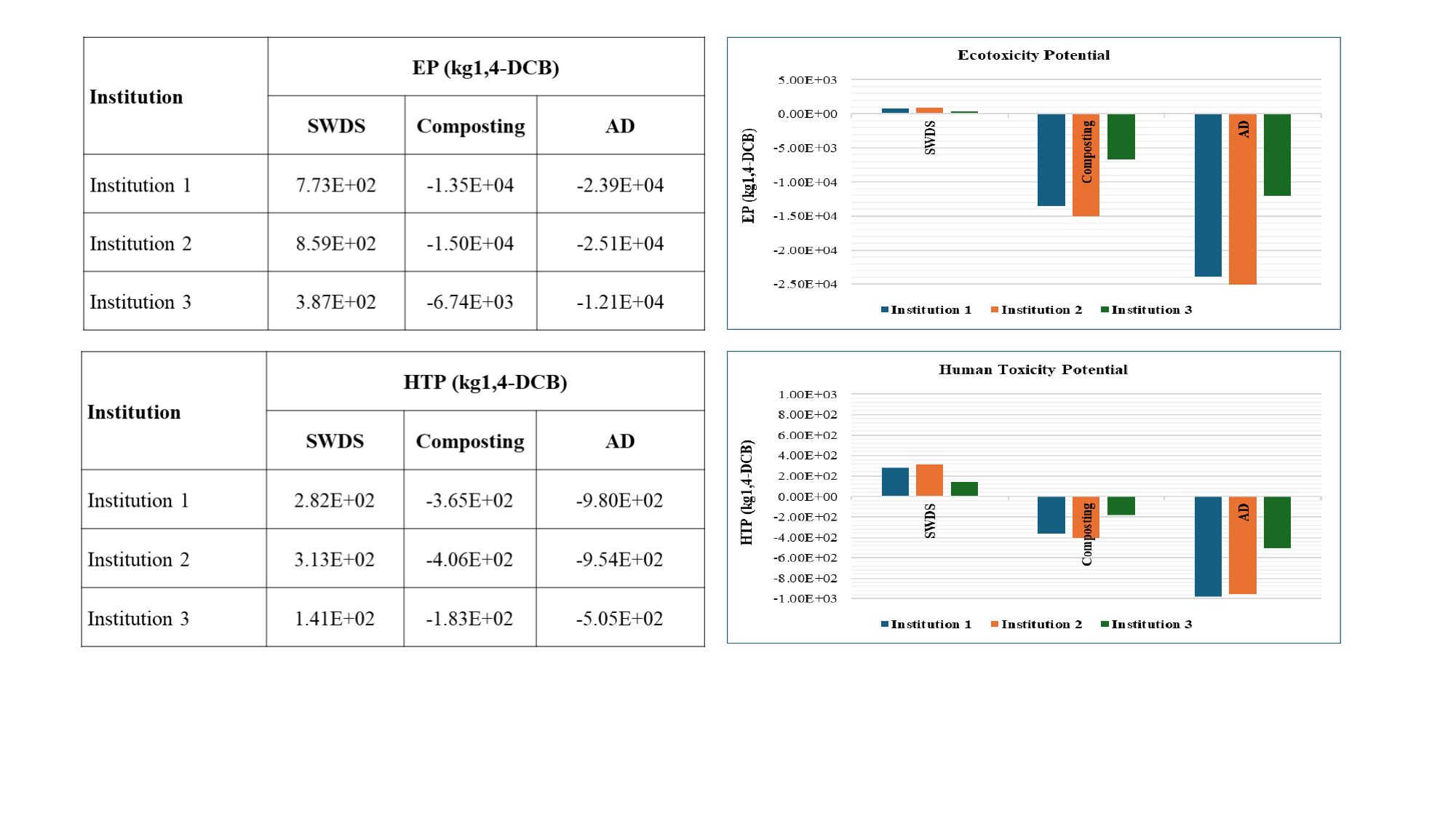

Supplement: Supplementary file 1 — Supplementary file1 (ZIP 861 KB) [file 10661_2024_13314_MOESM1_ESM.zip › EP and HTP Figure.pptx]

## Slide 1
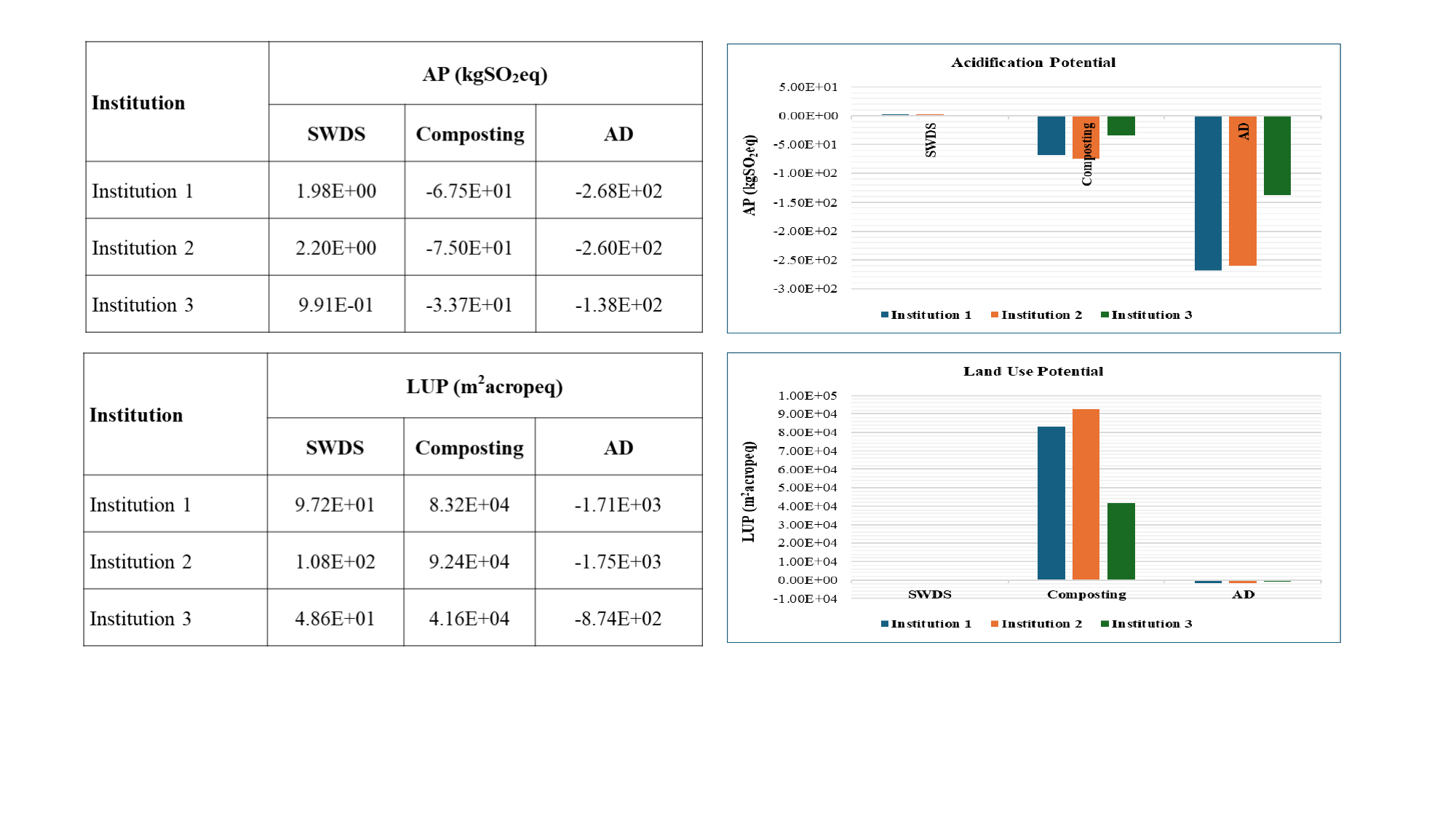

Supplement: Supplementary file 1 — Supplementary file1 (ZIP 861 KB) [file 10661_2024_13314_MOESM1_ESM.zip › FEP and MEP Figure.pptx]

## Slide 1
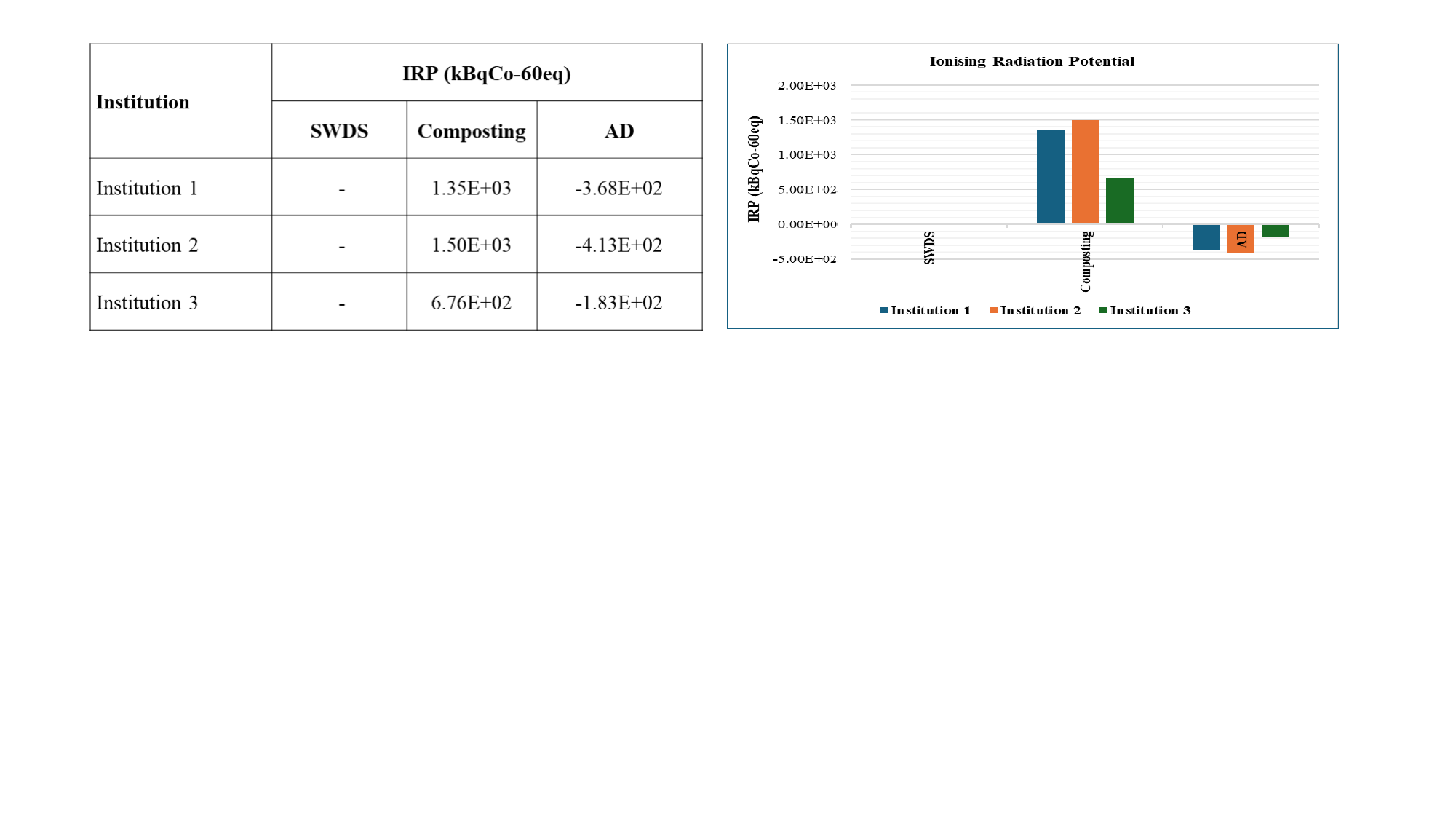

Supplement: Supplementary file 1 — Supplementary file1 (ZIP 861 KB) [file 10661_2024_13314_MOESM1_ESM.zip › IRP and FPMFP Figure.pptx]

## Slide 1
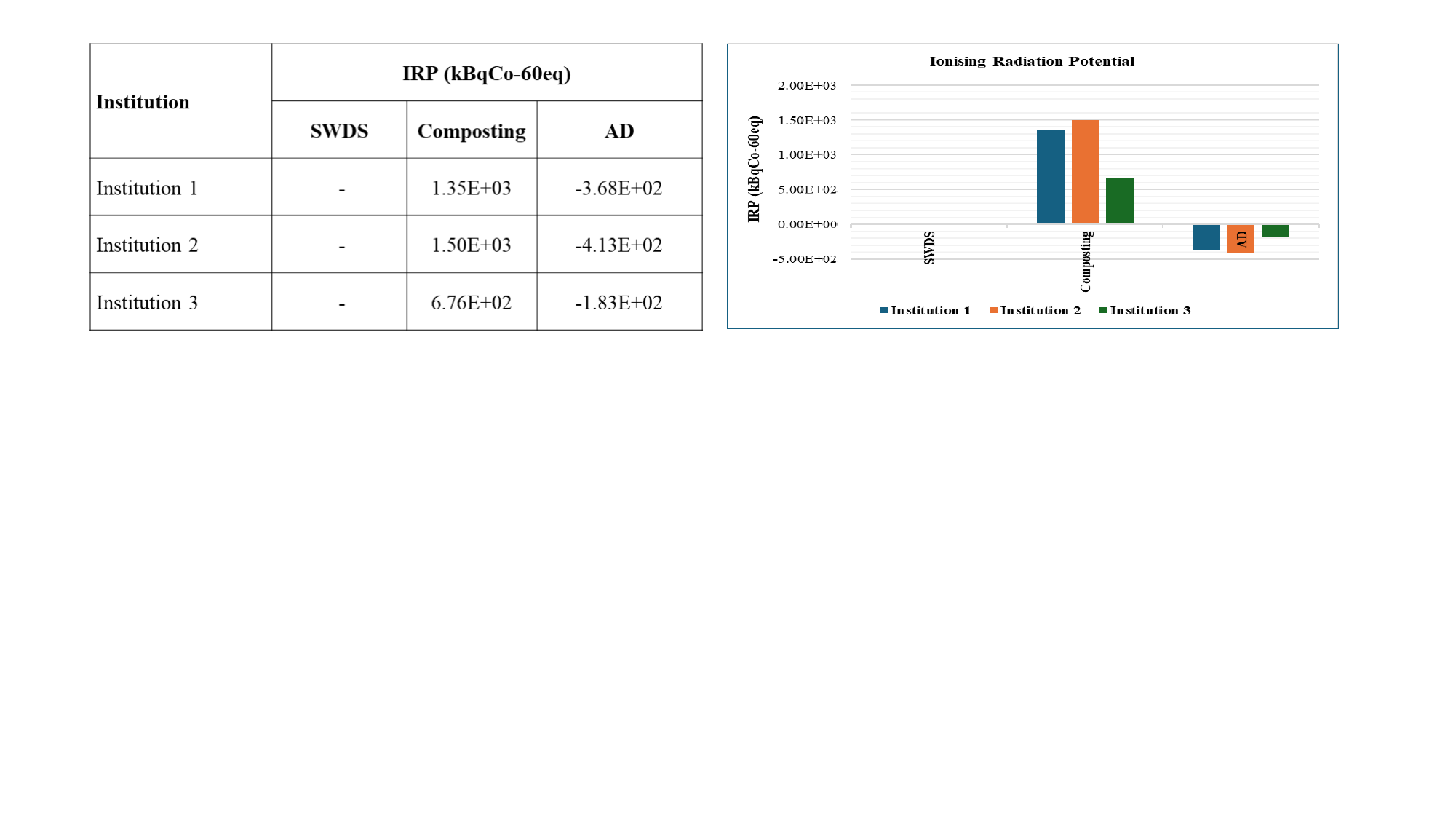

Supplement: Supplementary file 1 — Supplementary file1 (ZIP 861 KB) [file 10661_2024_13314_MOESM1_ESM.zip › OFP Figure.pptx]

## Slide 1
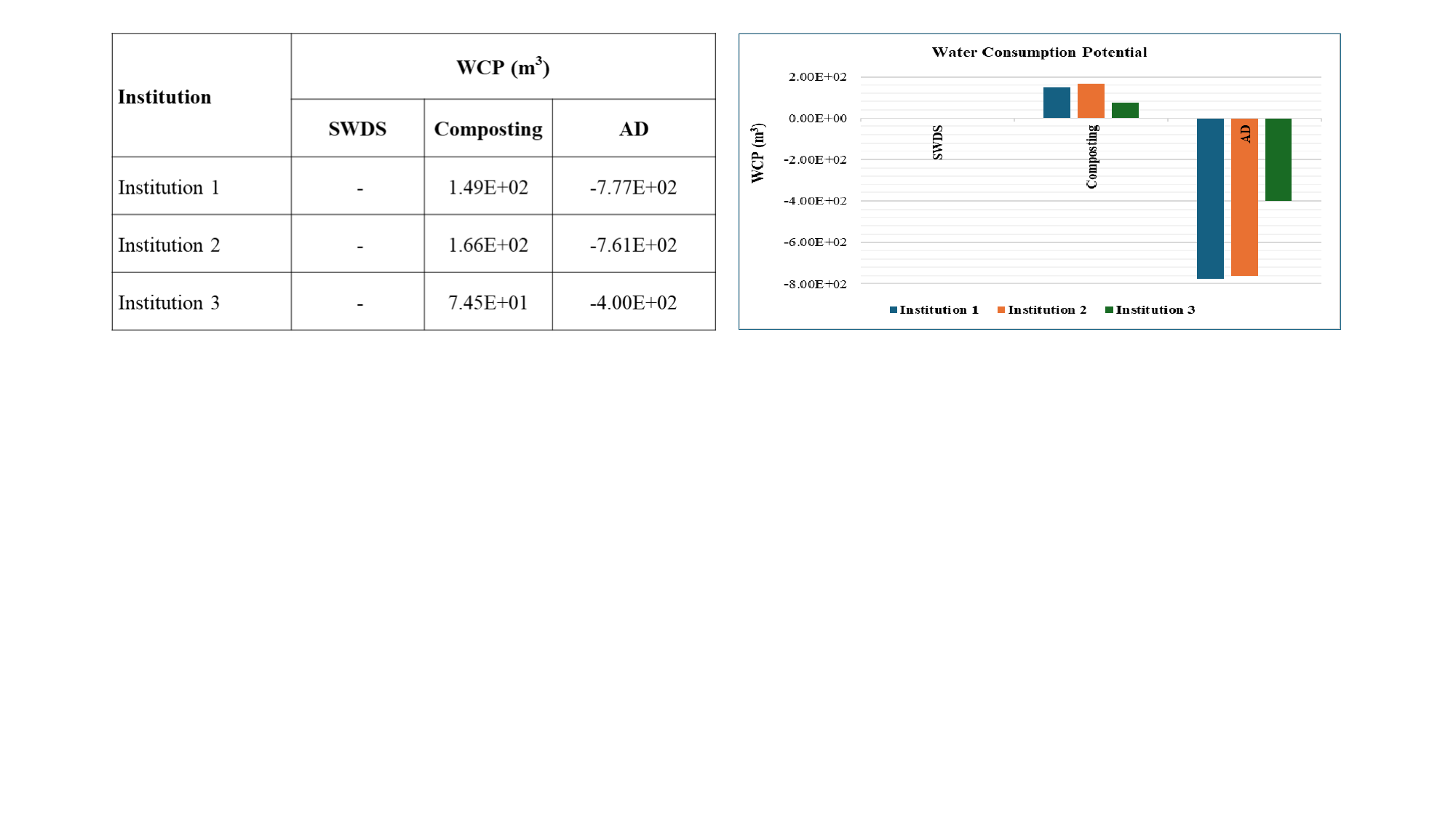

Supplement: Supplementary file 1 — Supplementary file1 (ZIP 861 KB) [file 10661_2024_13314_MOESM1_ESM.zip › WCP Figure.pptx]
